# Genome-wide identification and characterization of the carotenoid cleavage dioxygenase gene family in *Bixa orellana* L.

**DOI:** 10.3389/fpls.2026.1885876

**Published:** 2026-07-07

**Authors:** José Alberto Narváez-Zapata, Gabriel Garduño-Guadarrama, Diana Sierra-Ulín, Alejandra Cervera, Hugo Tovar, Renata Rivera-Madrid

**Affiliations:** 1 Instituto Politécnico Nacional, Centro de Biotecnología Genómica, Reynosa, Mexico; 2 Laboratorio de Genómica Biomédica y Bioinformática, Instituto Nacional de Medicina Genómica (INMEGEN), Mexico City, Mexico; 3 Unidad de Biología Integrativa, Centro de Investigación Científica de Yucatá, A.C. Mérida, Yucatán, Mexico; 4 Laboratorio de Biología de Sistemas Computacional y Genómica Integrativa, Instituto Nacional de Medicina Genómica (INMEGEN), Mexico City, Mexico

**Keywords:** achiote, annatto, *Bixa orellana*, bixin, CCD, cis-regulatory elements, gene family, genome-wide analysis

## Abstract

*Bixa orellana* L. is the primary source of the apocarotenoid bixin, whose biosynthesis is predicted to be initiated by carotenoid cleavage dioxygenases (CCDs). A genome-wide analysis was conducted to identify nonredundant CCD genes in *B. orellana*, classifying them into subfamilies. Gene structure, codon-based Ka/Ks divergence, conserved domains and motifs, subcellular localization, chromosomal distribution, paralog pairs, synteny, phylogenetic relationships (including reference orthologs from *A. thaliana* and *T. cacao*), transcriptomic expression patterns, and promoter cis-regulatory elements were all analyzed. These analyses identified 28 nonredundant CCD genes in *B. orellana*, classified into the CCD1, CCD4, CCD7, CCD8, NCED, and LCO subfamilies. Gene structure fell into three classes: intronless/single-exon genes (likely retrotransposition-derived), genes with intermediate 2–8 exon organizations, and complex genes with 10–15 exons (264 bp–6,254 bp genomic length). Ka/Ks analysis showed heterogeneous synonymous divergence (Ks: 0.367–1.449). Ten conserved sequence motifs were identified across all 28 proteins, including a broadly conserved NDH-type catalytic motif in 20 of 28 proteins spanning multiple subfamilies; full-length CCD1, CCD4, and NCED members harbored the complete motif set. Subcellular localization was predominantly cytoplasmic for CCD1 and core CCD4 members, with CCD1_copy3/4/5 predicted in peroxisomes and BoCCD4_chl isoforms in chloroplasts. Chromosomal mapping revealed two major hotspots: a CCD1 tandem array on scaffold 4 and a CCD4 cluster on scaffold 9, accounting for 59.4% of all CCD genes. Paralog analysis identified 39 tandem duplication candidate pairs (distance < 200 kb, Ks < 1.0), supported by synteny analysis showing partial flanking gene conservation (CCD1, CCD7, CCD8, NCED) and lineage-specific reorganization of the scaffold 9 CCD4 cluster. Phylogenetic analysis confirmed expansion of the CCD1 and CCD4 subfamilies, particularly CCD4-2 copies on scaffold 9. Transcriptomic data showed elevated seed expression of CCD1_copy5, CCD4-1, CCD4-3, and several CCD4-2 variants. Promoter analysis predicted abundant light-responsive elements, followed by hormone- and stress-responsive elements. These findings provide quantitative evidence that tandem duplication is the primary driver of CCD family expansion in *B. orellana*. The elevated seed expression of specific CCD1 and CCD4 copy variants is consistent with predicted roles in bixin biosynthesis, while the distinct subcellular localization patterns and abundant light-, hormone-, and stress-responsive promoter elements suggest functional diversification and regulatory complexity within the CCD gene family that may underlie pathway-specific apocarotenoid production.

## 1 Introduction

The seeds of the tropical shrub achiote (*Bixa orellana* L) or annatto (a term used for both the pigment and the plant) are the primary source of a natural pigment widely used in the food, cosmetic, and textile industries (Giuliano et al., 2003). This pigment is composed of water-soluble norbixin and oil-soluble bixin, with the latter being the most abundant apocarotenoid, accounting for over 80% of the total pigments in the seed (Raddatz-Mota et al., 2017; Rodríguez-Ávila et al., 2011; Cárdenas-Conejo et al., 2015). Despite its economic importance, researchers have not fully elucidated the biosynthetic pathway of bixin and its molecular regulation.

Bixin biosynthesis was demonstrated by Bouvier et al. (2003), who used a heterologous system in *Escherichia coli* to identify three key enzymes: a carotene-cleaving dioxygenase (*BoLCD or CCD*), a bixin aldehyde dehydrogenase (*BoADH or ALDH*), and a norbixin carboxylase methyltransferase (BonBMT or SABATH). However, to date, researchers have not fully reproduced this pathway with the proposed enzymes (Sergeant et al., 2009; Walter and Strack, 2011; Rodríguez-Ávila et al., 2011). Subsequent studies have identified other candidate genes associated with carotenoid and apocarotenoid metabolism in *B. orellana* including bixin; such as caffeic acid O-methyltransferase (*COMT*), 1-deoxy-D-xylulose-5-phosphate synthase (*DXS*), aldehyde dehydrogenase (*ALDH*), and members of the SABATH family of methyltransferases (Rodríguez-Ávila et al., 2011; Rivera-Madrid et al., 2016; Cárdenas-Conejo et al., 2015; 2023). A critical, established first step in bixin biosynthesis is the oxidative cleavage of lycopene at the 5,6/5’,6’ double bonds, a reaction catalyzed by carotenoid cleavage dioxygenases (*CCD*s) to yield bixin aldehyde (Carballo-Uicab et al., 2019; Us-Camas et al., 2022). In plants, the carotenoid cleavage oxygenase (*CCO*) enzyme family is divided into two main groups: the nine-cis-epoxycarotenoid dioxygenases (*NCED*s), pivotal in abscisic acid (ABA) biosynthesis, and the *CCD*s. The *CCD*s are further classified into subfamilies (*CCD1*, *CCD4*, *CCD7*, and *CCD*8) with distinct physiological roles. *CCD1* enzymes generate volatile apocarotenoid aromas like β-ionone; *CCD4* members are key determinants of color in flowers, fruits, and seeds; while *CCD7* and *CCD8* are involved in the synthesis of strigolactones, a class of apocarotenoid hormones that regulate plant architecture (Tan et al., 2003). In *B. orellana*, specific *CCD*s, including *BoCCD4-1*, *BoCCD4-3*, and *BoCCD1-1*, exhibit expression patterns that correlate with bixin accumulation during seed development. Functional characterization has confirmed that *BoCCD4–3* and *BoCCD1–1* can cleave lycopene *in vitro* at the 5,6-5’,6’ positions (Carballo-Uicab et al., 2019). More recently, a broader set of eight *BoCCD* genes has been implicated in the initial step of bixin biosynthesis, with several enzymes (*BoCCD1-3*, *BoCCD1-4*, *BoCCD4-1*, *BoCCD4-2*, and *BoCCD4-4*) demonstrated to produce bixin aldehyde from lycopene in both *in vivo* and *in vitro* assays (Us-Camas et al., 2022).

The recent advent of genome sequencing in numerous plant species has greatly facilitated the genome-wide identification and analysis of gene families involved in specialized metabolism, including apocarotenoid production (Zheng et al., 2021; Wang et al., 2025). The recent sequencing of the *B. orellana* genome (Lieberman et al., 2026) provides an unprecedented opportunity to analyze the *CCD* gene family in this species comprehensively. Therefore, the objectives of this study were to: (1) perform a genome-wide identification and characterization of the *CCD* gene family in *B. orellana*; (2) analyze their phylogenetic relationships, gene structures, and functional domains; (3) investigate the cis-regulatory elements in their promoter regions to infer transcriptional regulation; and (4) determine their genomic distribution and localization to elucidate evolutionary history. This systematic analysis lays a crucial foundation for future functional studies on the role of CCD genes in bixin biosynthesis. Furthermore, it identifies promising genetic targets for the biotechnological enhancement of bixin production in *B. orellana*.

## 2 Materials and methods

### 2.1 Identification of the *CCD* gene members in the genome of *B. orellana*

We obtained from the *Bixa orellana* reference genome (GCA_054491675.1; Lieberman et al., 2026) the annotation files. The genome spans 261 Mb, contains 57,106 genes, is assembled at the scaffold level, and represents the only available genome for the genus *Bixa*. We performed the identification of carotenoid cleavage dioxygenase (*CCD*) gene family members using a custom R v4.3.2 pipeline. The workflow included annotation mining, sequence extraction and translation. GFF3 annotations were analyzed with dplyr v1.1.4 and screened for “CCD” terms in functional descriptions. Additionally, a subsequent manual search for the terms “dioxygenase” and “NCED” was implemented to reinforce the analysis. Corresponding gene IDs were curated and cross-referenced with the GTF file to retrieve gene structure information, including genomic coordinates, strand orientation, and feature types. *CCD*-related features were exported as a structured GTF file and summarized in **Supplementary Table 1**.

We extracted nucleotide sequences of identified *CCD* genes FASTA file using seqinr v4.2.36 and processed with Biostrings v2.70.2. from the genome assembly. Sequences were trimmed to multiples of three nucleotides to preserve reading frames, aligned with msa v1.34.0 using the ClustalW algorithm, and translated into protein sequences using the standard genetic code, with ambiguous codons represented as “X”. Nucleotide and protein sequences were saved in FASTA format and used for downstream phylogenetic and functional domain analyses with ape v5.8.1 and phangorn v2.12.1.

### 2.2 Functional domain analysis and characterization of *CCD* genes

To validate the identity and functional classification of candidate CCD genes, we implemented a dual domain-analysis strategy combining local motif detection and Pfam database searches in R v4.3.2. Local motif analysis was performed using Biostrings v2.70.2 to identify conserved structural and catalytic features characteristic of CCD/RPE65 enzymes (Ohmiya, 2009). The local search targeted multiple conserved signatures including: (i) the iron-binding tetrad of histidine residues (four histidines coordinating Fe²^+^ cofactor), arranged in the pattern H[X]_20-40_H[X]_20-40_H[X]_20-40_H and the alternative His-Asp/Glu-His configuration; (ii) RPE65/CCD-specific motifs containing conserved glycine and aromatic residues near histidines (G[LIVM][X]_2_[FYW][X]_3-5_H) (Chander et al., 2012); (iii) hydrophobic tunnel regions essential for carotenoid substrate binding, characterized by stretches of hydrophobic residues flanking aromatic amino acids (Daruwalla et al., 2020); (iv) an asparagine-aspartate-histidine (NDH-type) motif (N[X]_2_–_4_D[X]_10_–_20_H), implemented as a search pattern based on conserved residues observed in structurally characterized CCD/NCED proteins (Messing et al., 2010; Daruwalla et al., 2020); this motif was initially targeted as a potential NCED-enriched signature, and (v) the dioxygenase catalytic signature combining aromatic, histidine, and acidic residues ([FYW]H[X]_2-4_[DE][X]_15-25_H) (Messing et al., 2010). Motif positions and matches were identified using regular-expression searches, and histidine content and density were calculated as additional indicators of *CCD* identity. Candidates were classified as high, medium, or low confidence based on the presence and combination of key motifs. Finally, to complement local motif detection, protein sequences were queried against the Pfam database using the HMMER hmmscan web API via the httr v1.4.7 package. Searches were monitored asynchronously, and results were parsed using jsonlite v1.8.8 to extract domain identities, coordinates, and statistical scores. Domain annotations were screened for *CCD*-relevant keywords (e.g., “RPE65,” “CCD,” “dioxygenase,” “NCED”), and proteins containing matching Pfam domains were designated high-confidence *CCD* candidates. A delay was applied to submissions to comply with API usage policies. Results from both approaches were integrated using dplyr v1.1.4 to generate a consolidated list of *CCD* candidates, prioritizing proteins supported by convergent evidence.

To complement the domain analysis, a conserved motif search was performed on the 28 curated *B. orellana* CCD protein sequences using the MEME Suite v5.5.9 (Bailey et al., 2015) with the following parameters: protein alphabet, ZOOPS model (zero or one occurrence per sequence), 10 motifs requested, minimum width of 6 aa, and maximum width of 50 aa, using the classic objective function with a zero-order Markov background model. Input sequences were provided in the tree order established by the maximum-likelihood phylogenetic analysis (Section 1.3) to facilitate downstream visualization. Both the MEME-derived motifs and the locally detected domain signatures (Fe_binding_1, Fe_binding_2, RPE65_motif_1/2/3, NDH-type motif, and Dioxygenase_signature) were extracted and visualized as scaled bar diagrams using a custom Python script (matplotlib v3.x), where each protein is represented as a proportional backbone scaled to its actual length in amino acids and each motif is displayed as a colored rectangle at its precise position, with sequences ordered to match the phylogenetic tree to enable direct comparison of conservation patterns across subfamilies.

### 2.3 Phylogenetic analysis of the *CCD* gene family

To examine the evolutionary relationships and phylogenetic classification of *CCD* genes in *B. orellana*, we performed a distance-based analysis was performed using translated protein sequences in R v4.3.2 with Biostrings, msa, and phangorn packages. Protein sequences of all *CCD* candidates were aligned using ClustalW, and evolutionary distances were estimated under the WAG amino acid substitution model, selected for its suitability in modeling enzyme family evolution (Whelan and Goldman, 2001). Sequence conservation across the alignment was assessed by calculating amino acid frequencies and gap occurrence, and by generating a position-specific conservation profile based on the most frequent residue at each site. Conservation patterns were visualized to identify highly conserved (80%) and variable (50%) regions within the *CCD* family.

Pairwise evolutionary distances were calculated to characterize divergence among *CCD* sequences, and a dendrogram was constructed from a hierarchical clustering of the normalized distance matrix. Pairwise evolutionary distances were calculated to characterize divergence among *CCD* sequences, and a dendrogram was constructed from a hierarchical clustering of the normalized distance matrix. To estimate true synonymous (Ks) and non-synonymous (Ka) substitution rates among the 28 curated *B. orellana* CCD genes, a codon-based Ka/Ks analysis was performed on the corresponding CDS nucleotide sequences. The protein alignment obtained with ClustalW was projected onto the CDS sequences using a custom pal2nal implementation in R, generating a codon-level alignment. Pairwise Ka and Ks values were then estimated for all 378 gene pairs using the Nei-Gojobori (NG86) method with Jukes-Cantor correction. Pairs where the proportion of synonymous differences exceeded the correction threshold (ps ≥ 0.75) returned undefined Ks values and were excluded from downstream comparisons; this is a known limitation of the NG86 method for highly divergent sequence pairs. Finally, to facilitate the association of *CCD* sequences identified in this study with previously characterized *BoCCD* genes for which functional activity has been experimentally demonstrated, additional pairwise protein alignments were performed between selected *CCD* sequences and their corresponding homologs from previously characterized *B. orellana* accessions exhibiting high sequence similarity. Sequence identity was calculated from ClustalW alignments as the proportion of identical amino acid positions relative to non-gap aligned positions, following standard alignment-based identity calculations.

### 2.4 Functional assignment

Putative orthologs of *B. orellana CCD* genes were identified using a custom dplyr-based pipeline that ranked reference sequences according to pairwise sequence identity. For each query, the three highest-scoring reference sequences from *B. orellana*, *Arabidopsis thaliana*, and *Theobroma cacao* were retrieved, and the top-ranking match was assigned as the best ortholog for functional annotation transfer. Subcellular localization was predicted using WoLF PSORT (https://wolfpsort.hgc.jp/) and incorporated into the annotation framework. Complete ortholog tables were generated for reference, along with a simplified best-match table for downstream analyses. Orthology assignments were further supported by phylogenetic reconstruction using the Neighbor-Joining method based on WAG distances, visualized with ggtree v3.10.1 and species-specific color coding to highlight orthologous relationships and lineage-specific expansions. In addition, a focused similarity heatmap of best ortholog matches was generated using pheatmap v1.0.13 to facilitate identification of high-confidence one-to-one orthologs and potential co-orthologs.

### 2.5 Orthology analysis and comparative phylogenomics

To infer evolutionary relationships between *B. orellana CCD* genes and characterized homologs, an orthology analysis was performed using protein sequences from *A. thaliana* (model dicot), *T. cacao* (phylogenetically related Malvales order species), and additional *B. orellana* entries available in public databases. Reference *CCD* proteins were retrieved from UniProt via the REST API using the term “Carotenoid cleavage dioxygenase”, yielding 21 sequences from *B. orellana*, 32 from *A. thaliana*, and 23 from *T. cacao*. Retrieved sequences were quality-checked for length (<300 amino acids) and completeness and combined with genome-derived *B. orellana* CCD proteins to generate a comparative dataset. Global sequence similarity was assessed using hierarchical clustering based on WAG distances and visualized as a similarity heatmap (pheatmap v1.0.13). Best orthologs for each *B. orellana CCD* gene were identified based on maximum sequence identity and validated by reciprocal best-hit analysis. Orthologous relationships were further visualized using a focused similarity heatmap comparing *B. orellana CCD* genes with reference sequences. For phylogenetic inference, a curated alignment containing *B. orellana CCD* genes and their confirmed subfamily representatives was used to construct a maximum-likelihood tree. The resulting phylogeny was visualized as a circular cladogram using the ape v5.8.1 and ggplot2 v3.x packages using the Most Recent Common Ancestor (MRCA) algorithm, with tips annotated by subfamily (*CCD1*, *CCD4*, CCD7, *CCD8*, *LCO*, and *NCED*) through color-coded symbols indicating both subfamily identity (fill color) and species of origin (symbol shape: circle for *B. orellana*, square for *A. thaliana*, triangle for *T. cacao*). Reference sequences with ambiguous phylogenetic placement or lacking experimental functional support were excluded from the final visualization to maximize clarity of subfamily assignment. Branch support was assessed by bootstrap analysis (1000 replicates), providing an integrated phylogenomic framework for *CCD* gene evolution in *B. orellana*.

### 2.6 Gene structure analysis of *CCD* family members

To characterize the genomic architecture and exon–intron organization, the filtered GTF of the selected *CCD* genes in *B. orellana* was used with the online GSDS version 2.0 (Bo et al., 2015) platform. A maximum-likelihood tree obtained from curated CCD protein alignment was used to complete the analysis. In addition, conserved protein motifs were identified using the GLAM2 algorithm implemented in the MEME Suite, which enables detection of semi-conserved motifs with insertions or deletions, providing a flexible representation of conserved functional regions within CCD proteins (Frith et al., 2008).

### 2.7 Promoter analysis and identification of cis-regulatory elements

To explore predicted transcriptional regulation of *CCD* genes in *B. orellana*, we conducted promoter analyses to identify cis-acting regulatory elements in upstream regions. Gene structure information was obtained from the filtered CCD GTF annotation using the GenomicFeatures package (part of Bioconductor v3.18), enabling the extraction of gene boundaries, exon organization, and coding features. Structural attributes, including genomic location, strand orientation, gene length, total exon length, and exon number, were summarized to characterize the *CCD* gene family. Promoter regions were defined as 2 kb upstream of the transcription start site for each gene, with the transcription start site (TSS) position determined based on strand orientation. Promoter coordinates were adjusted to ensure valid genomic boundaries, and promoter sequences were subsequently extracted from the *B. orellana* genome assembly for downstream cis-element identification.

### 2.8 Cis-regulatory element prediction and functional annotation

We analyzed promoter sequences using the PlantCARE database to identify cis-acting regulatory elements and their associated functions (Lescot et al., 2002). Identified motifs were filtered to remove ambiguous or unannotated entries, and a curated set of biologically relevant elements was selected based on their documented roles in plant gene regulation. This priority list included: stress-responsive elements (ABRE for abscisic acid response, MBS/MBSI for drought response, LTR for low-temperature response, TC-rich repeats for defense and stress, ARE for anaerobic induction); hormone-responsive elements (ERE for ethylene response, CGTCA-motif and TGACG-motif for MeJA response, TCA-element for salicylic acid response, P-box and GARE-motif for gibberellin response); light-responsive elements (G-box, Box 4, GT1-motif, AE-box, MRE); and developmental/circadian regulation elements (circadian, TCT-motif, WUN-motif). Each cis-element was classified into functional categories according to its annotation, and summary statistics were generated for each *CCD* gene, including the abundance and diversity of high-interest regulatory elements. All identifications are based on sequence similarity to known motifs in the PlantCARE database and represent predicted regulatory potential inferred from promoter sequence features; they do not constitute experimental evidence of transcriptional activity, transcription factor binding, or regulatory causality.

To visualize the distribution and abundance of regulatory elements across *CCD* genes, two complementary approaches were implemented. First, a dot plot was generated using ggplot2 v4.0.0 to display the frequency of high-interest motifs across all *CCD* genes. In this visualization, genes are arranged on the x-axis, individual motif types on the y-axis, dot colors represent functional categories, and dot sizes indicate the frequency of each motif per gene. Second, a circular Circos-style plot was constructed using the circlize and ComplexHeatmap packages to provide an integrative view of the predicted regulatory elements among genes, promoter positions, and regulatory element functions. The outer track of the circular plot shows individual *CCD* genes, arranged and color-coded by subfamily (*CCD1*, *CCD4*, *CCD4-chl*, *CCD7*, *CCD8*, *NCED*, and *LCO*). Each gene sector shows the relative positions of regulatory elements along the 2,000 bp promoter region (represented as negative kilobase values relative to the TSS). Regulatory elements are represented as colored points within each gene sector, with colors corresponding to functional categories: green for Light-responsive elements, deep pink for Stress development elements, dark gray for Hormone-responsive elements, and cyan for general Regulator elements. Curved links connect genes sharing similar promoter element composition within each functional category and should be interpreted as indicators of motif profile similarity only, not as evidence of functional co-regulation or shared transcriptional control. Link generation employed a frequency-based filtering approach using dplyr v1.1.4. For each functional category, genes were ranked by the abundance of elements in that category, and links were drawn between genes exceeding the 75th percentile of motif frequency, a conservative threshold selected to highlight genes with the highest relative enrichment of elements within each category while limiting visual overcrowding. For the Regulator category, all genes were included given the lower number of elements in this group. Hormone-responsive links (gray) were drawn first to serve as a background layer, with other categories overlaid in decreasing order of specificity.

### 2.9 Chromosomal distribution and genomic localization of *CCD* genes

To allow comparison across scaffolds of different lengths, gene positions were normalized as percentages of total scaffold length using scaffold size information from the ten largest scaffolds, which comprise most of the assembled genome. Normalized positions were calculated as (gene start/scaffold length) × 100. This approach allows direct comparison of gene distribution patterns across scaffolds regardless of their absolute sizes. To ensure uniform visual height across all scaffolds in the final figure, particularly for scaffolds carrying only one or two genes, where min and max positions would otherwise collapse to a single point, anchor markers were added at positions 0% and 100% of each scaffold prior to plotting; these carry blank labels and do not represent real gene loci. A linkage map-style visualization was generated with LinkageMapView to display *CCD* gene distribution on the four most gene-dense scaffolds (4, 5, 9, and 10). Scaffolds 1 and 6, each carrying a single CCD gene, were excluded from the visualization as they do not contribute meaningfully to the analysis of gene clustering patterns. To improve label readability in regions of high gene density, a minimum positional offset of 1.5% of scaffold length was applied between adjacent gene labels in scaffolds 4 and 9, where tandem duplication events have produced multiple physically proximate gene copies. This offset affects only label positioning and does not alter the underlying genomic coordinates, which are provided in **Supplementary Table 1**.

### 2.10 Transcriptomic analysis of *CCD* gene expression

To investigate the expression patterns of *CCD* genes across different tissues, we conducted a transcriptomic analysis using publicly available RNA-seq data. For this, transcript sequences from the *B. orellana* Transcriptome Shotgun Assembly (TSA, BioProject PRJNA895001) were retrieved and used as a reference database (Cárdenas-Conejo et al., 2015). A local protein BLAST database was built from the TSA using the rBLAST package (v0.99.2). The protein sequences of the identified *BoCCD* genes were queried against this database using tBLASTn with default parameters. The resulting hits were processed to assign the best match for each query based on percentage identity, alignment length, and E-value, following a custom function implemented in dplyr v1.1.4. The final best-hit assignments were exported for downstream analysis. To quantify expression levels, a counts table was constructed from raw RNA-seq data for leaf and seed tissues from the P12 accession of *B. orellana*, previously submitted under BioSample accessions SAMN31488112-SAMN31488147 (Cárdenas-Conejo et al., 2023). Counts were normalized using the edgeR package (v3.40.2) to obtain log_2_-transformed counts per million (logCPM). A heatmap was generated using the gplots package (v3.1.3) to visualize expression patterns across tissues, with a color palette from RcolorBrewer (v1.1.3) to highlight differential expression.

### 2.11 Synteny analysis of CCD gene subfamilies

To assess the conservation of genomic context across species, a synteny analysis was conducted for each *CCD* subfamily identified in *B. orellana*. For each subfamily, the representative gene with the highest ortholog identity was selected as the focal gene. Flanking genomic windows of five genes on each side of each focal *B. orellana CCD* locus were extracted from the full AUGUSTUS/BRAKER annotation (achiote_braker.gtf) using a custom R v4.3.2 pipeline. Gene-level coordinates were derived from transcript feature rows, retaining the longest isoform per locus. The best-matched ortholog for each subfamily in *A. thaliana* (TAIR10) and *T. cacao* (Phytozome v13), identified in Section 1.4, was used as the reference anchor, and a window of equivalent flanking genes was retrieved for each reference species. Synteny plots were generated using the gggenes v0.5.1 package, with gene arrows colored by gene class (focal *B. orellana CCD*, adjacent *B. orellana CCD*, best ortholog, or neighboring gene) and faceted by genomic track per species. Genomic positions were expressed in base pairs and oriented relative to the focal gene strand. Plots were generated independently for each subfamily (*CCD1*, *CCD4*, *CCD7*, *CCD8*, *NCED*, and *LCO*) and saved as individual PDF/PNG files using ggplot2 v4.0.0 and patchwork v1.2.0.

### 2.12 Formal paralog pair analysis of tandem duplication

To provide formal evidence for tandem duplication as the primary driver of *B. orellana CCD* family expansion, a paralog pair analysis was conducted combining genomic distance, synonymous substitution rates, and physical co-localization criteria. For each of the 378 pairwise comparisons among the 28 curated *CCD* genes, genomic coordinates were retrieved from the filtered GTF annotation (**Supplementary Table 1**) and midpoint positions were calculated for each gene. Pairwise genomic distances were computed for all same-scaffold gene pairs (n = 116). A paralog pair was classified as a tandem duplication candidate if it simultaneously met three criteria: (i) location on the same scaffold, (ii) genomic distance between gene midpoints < 200 kb, a threshold consistent with standard tandem duplication definitions applied in plant genome studies (Zhou et al., 2019, 2020), and (iii) mean pairwise Ks < 1.0, excluding pairs where synonymous site saturation precluded reliable estimation. Pairwise Ka and Ks values were obtained from the codon-based analysis described in Section 1.3. Tandem arrays were further characterized by calculating consecutive inter-gene distances along each scaffold.

## 3 Results

### 3.1 Structural diversity and genomic organization of *CCD* genes in *B. orellana*

*CCD* gene identification followed this flowchart: (i) Automated mining and manual confirmation in the genome = 34 putative *CCD* members. (ii) Local motif detection and Pfam-based domain annotation = 32 high-confidence *CCD* candidates. (iii) Phylogenetic analysis and ortholog identity filter (≥30%) = 28 non-redundant *CCD* genes. First structural analysis of *CCD* gene family in *B. orellana* revealed 34 putative *CCD* members, prior to motif detection and Pfam-based domain annotation, distributed across four major scaffolds (**Table 1**). Complete genomic annotations for all identified *CCD* genes, including scaffold assignments, feature types (transcript, exon, CDS, intron, start and stop codons), precise genomic coordinates, strand orientation, and attribute fields, are documented in **Supplementary Table 1**. These annotations, derived from AUGUSTUS gene predictions in the GTF format, served as the foundation for extracting structural metrics and informing downstream phylogenetic and functional analyses. The 34 putative *CCD* members exhibited substantial architectural diversity in terms of genomic organization and exon-intron structure (**Table 1**). Genes were unevenly distributed across six scaffolds: ptg000001l (n=1), ptg000004l (n=6), ptg000005l (n=9), ptg000006l (n=1), ptg000009l (n=13), and ptg000010l (n=4).

**Table 1:** Structural characteristics of *CCD-*related genes in *B. orellana*.

Scaffold	Gene id	Tx name	Start	End	Strand	Tx length	Exon count	Intron count	Total exon length	Total intron length	Total exon length kb	Number of exons	Transcripts
ptg000001l	g2824	g2824.t1	14807234	14808985	–	1752	1	0	1752	0	1.752	1	1
ptg000004l	g17625	g17625.t1	10960055	10966186	+	6132	14	13	1785	4347	1.785	14	1
ptg000004l	g17629	g17629.t1	10982806	10989059	+	6254	14	13	1686	4568	1.686	14	1
ptg000004l	g17630	g17630.t1	10994673	11000312	+	5640	15	14	1758	3882	1.758	15	1
ptg000004l	g17631	g17631.t1	11005955	11011656	+	5702	14	13	1629	4073	1.629	14	1
ptg000004l	g17637	g17637.t1	11039020	11044844	+	5825	14	13	1629	4196	1.629	14	1
ptg000004l	g19004	g19004.t1	17220196	17221752	+	1557	1	0	1557	0	1.557	1	1
ptg000004l	g19008	g19008.t1	17232272	17233828	+	1557	1	0	1557	0	1.557	1	1
ptg000005l	g23079	g23079.t1	8581661	8586788	+	5128	14	13	1644	3484	1.644	14	1
ptg000005l	g24578	g24578.t1	17184566	17188114	–	3549	11	10	1809	1740	1.809	11	1
ptg000005l	g24580	g24580.t1	17189504	17193860	–	4357	12	11	1827	2530	1.827	12	1
ptg000005l	g24581	g24581.t1	17197106	17198732	–	1627	4	3	702	925	0.702	4	1
ptg000005l	g24582	g24582.t1	17202270	17204934	–	2665	8	7	1200	1465	1.2	8	1
ptg000005l	g24585	g24585.t1	17210320	17211122	–	803	3	2	360	443	0.36	3	1
ptg000005l	g24592	g24592.t1	17278394	17281683	+	3290	6	5	1692	1598	1.692	6	1
ptg000005l	g25911	g25911.t1	23859237	23861258	–	2022	2	1	1683	339	1.683	2	1
ptg000006l	g31486	g31486.t1	18933032	18935506	+	2475	2	1	1959	516	1.959	2	1
ptg000009l	g33764	g33764.t1	10675286	10675704	–	419	2	1	336	83	0.336	2	1
ptg000009l	g33765	g33765.t1	10676480	10677409	–	930	3	2	492	438	0.492	3	1
ptg000009l	g37259	g37259.t1	28742182	28743765	+	1584	4	3	876	708	0.876	4	1
ptg000009l	g37262	g37262.t1	28753578	28755152	+	1575	1	0	1575	0	2.016	2	2
ptg000009l	g37262	g37262.t2	28754712	28755152	+	441	1	0	441	0	2.016	2	2
ptg000009l	g37263	g37263.t1	28757043	28758722	–	1680	1	0	1680	0	3.069	3	2
ptg000009l	g37263	g37263.t2	28757254	28758722	–	1469	2	1	1389	80	3.069	3	2
ptg000009l	g37264	g37264.t1	28760572	28760910	–	339	1	0	339	0	0.963	4	2
ptg000009l	g37264	g37264.t2	28759540	28760910	–	1371	3	2	624	747	0.963	4	2
ptg000009l	g37265	g37265.t1	28765475	28767043	–	1569	1	0	1569	0	1.569	1	1
ptg000009l	g37266	g37266.t1	28766212	28767043	–	832	2	1	753	79	0.753	2	1
ptg000009l	g37268	g37268.t1	28785508	28787076	–	1569	1	0	1569	0	1.569	1	1
ptg000009l	g37269	g37269.t1	28789558	28791745	+	2188	3	2	447	1741	0.447	3	1
ptg000009l	g37270	g37270.t1	28794383	28796155	–	1773	1	0	1773	0	1.773	1	1
ptg000010l	g37989	g37989.t1	6659491	6659754	–	264	1	0	264	0	0.264	1	1
ptg000010l	g38864	g38864.t1	11031543	11034071	–	2529	6	5	1854	675	1.854	6	1
ptg000010l	g39248	g39248.t1	12686919	12692776	+	5858	14	13	1644	4214	1.644	14	1

The marked clustering of 13 genes (38%) on scaffold ptg000009l suggests a potential tandem duplication hotspot or a genomic region enriched in *CCD* family expansion. Gene widths ranged from 264 bp (g37989) to 6,254 bp (g17629), reflecting a 23.7-fold difference in genomic span. No UTR features were detected in any of the genes based on the current annotations. Total coding exon lengths varied from 0.264 kb to 1.959 kb, with exon numbers ranging from 1 to 15 per gene. Structural analysis revealed two distinct architectural classes within the *B. orellana CCD* family. A subset of genes (g2824, g19004, g19008, g37262, g37263, g37265, g37268, g37270, g37989) consisted of single exons, potentially indicating retrotransposition-derived intronless paralogs or evolutionary recent duplications that have not yet acquired introns. In contrast, structurally complex multi-exon genes, particularly those containing 14–15 exons (g17625, g17629, g17630, g17631, g17637, g23079, g39248), exhibited canonical plant *CCD* gene architecture comparable to well-characterized *CCD1* and *CCD4* subfamily members in *A. thaliana*. An intermediate group of genes displayed moderate complexity with 2–8 exons such as g24582 (8 exons), g38864 (6 exons), g24592 (6 exons), g31486 (2 exons), g25911 (2 exons), g33764 (2 exons), g33765 (3 exons), g37259 (4 exons), g37264 (up to 4 exons), g37266 (2 exons), and g37269 (3 exons), suggesting diverse evolutionary trajectories within the family. Strand orientation analysis showed genes distributed across both positive (n=16) and negative (n=13) strands, with no apparent correlation between strand preference and scaffold location or structural complexity. The variation in exon and intron architecture across the *CCD* family likely reflects a combination of functional constraint on catalytic domains and lineage-specific diversification, potentially associated with specialized roles in bixin biosynthesis and other carotenoid-derived metabolic pathways in *B. orellana*.

### 3.2 Functional domain and motif compositions of predictable CCD proteins

The dual-approach analysis, integrating local motif detection and Pfam-based domain annotation, supported the structural and catalytic identities of the high-confidence *CCD* candidates. In total, 32 predictable proteins were classified as high-confidence *CCD* candidates, all of which contained one or more characteristic dioxygenase signatures. The local motif search detected a total of 141 motif instances across these sequences, encompassing five conserved categories: iron-binding histidine tetrads, dioxygenase catalytic signatures, RPE65-type motifs, NDH-type (asparagine-aspartate-histidine) motifs, and hydrophobic tunnel-associated patterns (**Supplementary Table 2**). The iron-binding histidine tetrad (H[X]_20_–_40_H[X]_20_–_40_H[X]_20_–_40_H) or its shortened variants were present in all analyzed sequences, typically located between residues 100 and 500. These motifs correspond to the conserved Fe²^+^ coordination sites essential for catalysis. The dioxygenase catalytic signature ([FYW]H[X]_2_–_4_[DE][X]_15_–_35_H) was detected in 30% of the proteins, suggesting their functional identity as non-heme iron oxygenases. RPE65/CCD-specific motifs (G[LIVM][X]_2_[FYW][X]_3_–_5_H and P[X]_2_–_4_[LIVM][X]_2_–_4_H) were the most frequently recovered, often clustered around residues 200–250 and 450–500. These aromatic-rich motifs flank hydrophobic regions associated with substrate channels and are consistent with the presence of extended hydrophobic tunnel stretches observed in structural analogs (Chander et al., 2012; Daruwalla et al., 2020). The NDH-type motif (N[X]_2_–_4_D[X]_10_–_20_H) was detected in 20 of the 28 curated proteins, distributed across multiple subfamilies (12 CCD4, 5 CCD1, 2 NCED, and 1 LCO member), indicating that this is a broadly conserved structural signature within the CCD/RPE65 superfamily rather than an NCED-exclusive feature. Histidine density analysis showed that CCD proteins contained 1.36–3.85% histidine residues. The integrated scoring system classified 27 sequences (79.4%) as high-confidence CCDs that met both the iron-binding and RPE65-motif criteria, and 5 (14.7%) as low-confidence candidates with partial motif conservation. Two proteins were discarded at this stage due to the absence of some CCD-related domains (g24585.t1 and g33764.t1). Pfam analysis further validated these results, identifying significant matches (E-values ranging from 1.2×10^-^³^0^ to 4.6×10^-^¹^6^) to the domains PF03055 (RPE65 family), PF10543 (Carotenoid oxygenase), and PF05270 (Dioxygenase/NCED-like). In summary, integrating motif- and domain-level analyses provided convergent molecular evidence for classifying these proteins as probable members of the carotenoid cleavage dioxygenase family. The positional distribution of all detected motif types across the 28 curated CCD proteins, including iron-binding histidine tetrads, RPE65-specific motifs, NDH-type signatures, and the dioxygenase catalytic signature, is presented in **Supplementary Figure 4**, ordered according to the maximum-likelihood phylogenetic tree. This visualization confirms that full-length members of the CCD1, CCD4, and NCED subfamilies consistently harbor the broadest and most spatially conserved domain repertoire, while structurally simpler or divergent sequences such as g33764 and g37269 show absent or minimal domain coverage, consistent with their truncated or non-canonical protein structures. The high degree of conservation across catalytic, aromatic, and hydrophobic motifs underscores the evolutionary stability of the CCD/RPE65 structural framework. In contrast, the broader distribution of the NDH-type motif across CCD1, CCD4, LCO, and NCED subfamilies suggests that this signature reflects a shared structural feature of the CCD/RPE65 fold rather than a marker of subfunctionalization exclusive to the NCED lineage.

### 3.3 Orthology relationships of the C*CD*-related genes

**Figure 1:**
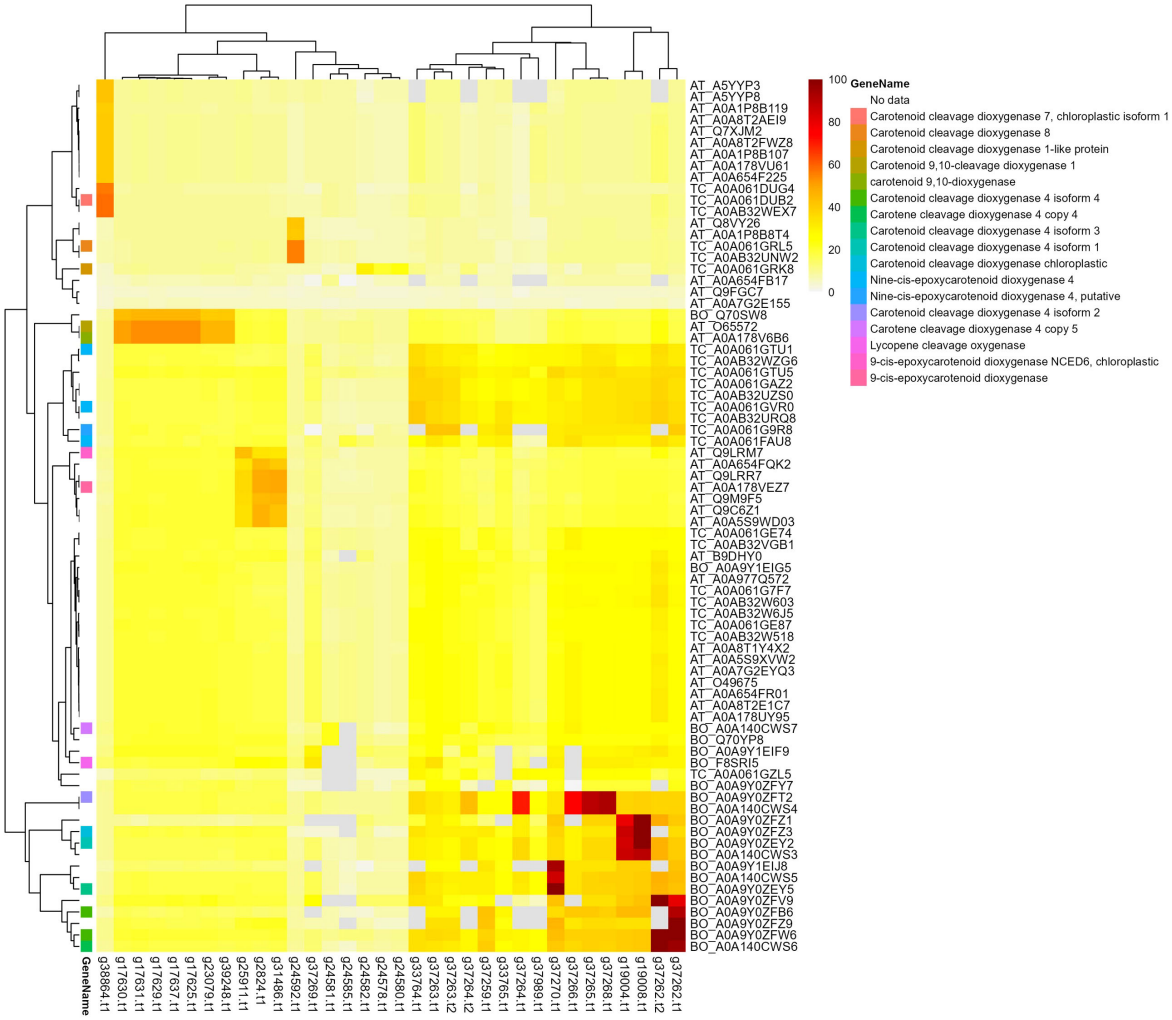
Orthology assignment of *B. orellana CCD-*related genes. Targeted similarity heatmap focusing on *B. orellana CCD* genes versus their best-matched orthologs from reference species. Column shows reference sequences annotated with gene names from UniProt, no data indicates an unclear annotation, row shows *B. orellana* query sequences. The heatmap highlights specific orthologous relationships used for functional annotation transfer.

To elucidate the evolutionary relationships and functional classification of related *CCD* genes, we conducted a comparative phylogenomic analysis, incorporating 76 CCD protein sequences from three species: *B. orellana* (21), *A. thaliana* (32), and *T. cacao* (23). Quality control assessment of the retrieved sequences revealed lengths ranging from 312 to 612 amino acids (mean = 534 aa, median = 548 aa), with all sequences meeting the minimum threshold of 300 amino acids, indicating complete or near-complete protein annotations suitable for phylogenetic inference. First, an initial pairwise sequence identity matrix was computed for all 110 (*CCD*-related and orthologous genes) *CCD* sequences using the identity_matrix method, followed by hierarchical clustering to reveal global conservation patterns across the *CCD* family. The pairwise sequence identity heatmap (**Supplementary Figure 1**) reveals a clear functional partitioning of the analyzed sequences into four major clades, consistent with the known evolutionary and functional divergence within the carotenoid cleavage dioxygenase (*CCD*) superfamily. Clade I, the largest group, occupies the central-upper diagonal block and encompasses most sequences annotated as *CCD4*. This clade includes the five *B. orellana CCD4* paralogs (A0A140CWS3–7), multiple additional *B. orellana* isoforms (A0A9Y0ZFT2, ZFW6, ZFZ3, among others), and the sequences annotated as “Probable *CCD4*, chloroplastic” from *T. cacao* (A0AB32URQ8, UZS0, VGB1, W518, W603, W6J5), reflecting high intragroup sequence conservation. Clade II forms a distinct middle block comprising the *CCD7* orthologs (AT_Q7XJM2, MAX3 fragments A5YYP3/A5YYP8, A0A1P8B107/B119) and *CCD8* sequences (AT_Q8VY26, A0A1P8B8T4, TC_A0A061GRL5, TC_A0AB32UNW2), which co-cluster with high internal similarity, in agreement with their shared role in the strigolactone biosynthesis pathway. Clade III, resolved in the lower-right corner of the heatmap, groups the 9-*cis*-epoxycarotenoid dioxygenases (*NCEDs*) involved in abscisic acid biosynthesis, including *A. thaliana NCED3* (Q9LRR7), *NCED5* (Q9C6Z1), *NCED6* (Q9LRM7), and *NCED9* (Q9M9F5), together with putative *NCED4* sequences from *T. cacao* (A0A061G7F7, GE87, FAU8, G9R8, GAZ2, GE74, GTU1, GTU5, GVR0) and several hypothetical *Arabidopsis* entries. Finally, Clade IV, located in the lower-middle region, co-groups the *B. orellana* lycopene cleavage oxygenase sequences (BO_Q70YP8 and BO_F8SRI5) with *A. thaliana CCD1* (AT_O65572), consistent with the known evolutionary proximity between plant *CCD1* enzymes and lycopene cleavage oxygenases, both of which act on linear carotenoid substrates, despite differences in their precise cleavage positions (9,10/9′,10′ for *AtCCD*1 versus 5,6/5′,6′ for *BoLCO*). To identify the most closely related orthologs for each *B. orellana CCD* gene, the identity matrix was filtered to extract query-versus-reference comparisons exclusively (**Supplementary Table 3**). A refined heatmap was generated displaying only *B. orellana* queries against their respective best orthologs (**Figure 1**). The heatmap used a gradient color scheme (white-yellow-orange-red-dark red) to represent identity percentages, with row annotations showing the Gene Names of the best orthologs. The heatmap revealed a clear functional organization across the horizontal dendrogram. Four major clades were distinguished based on sequence identity. The leftmost clade grouped sequences with similarity to *CCD1* and lycopene cleavage oxygenases from *B. orellana* and *A. thaliana*. The adjacent clade clustered *CCD7* and *CCD8* orthologs from *A. thaliana* and *T. cacao*, consistent with their shared role in strigolactone biosynthesis. A central clade encompassed *NCED* members from *A. thaliana* and *T. cacao*, which are associated with abscisic acid (ABA) biosynthesis. Notably, the rightmost clade showed strong and exclusive similarity to *CCD4* sequences from *B. orellana*, including multiple gene copies (*CCD4–1* through *CCD4-6*) previously linked to bixin and norbixin biosynthesis. This clustering pattern reflects the known functional divergence within the *CCD*/*NCED* family and supports the classification of the query sequences into functionally distinct subfamilies.

### 3.4 Subfamily assignment of the C*CD*-related genes

**Figure 2:**
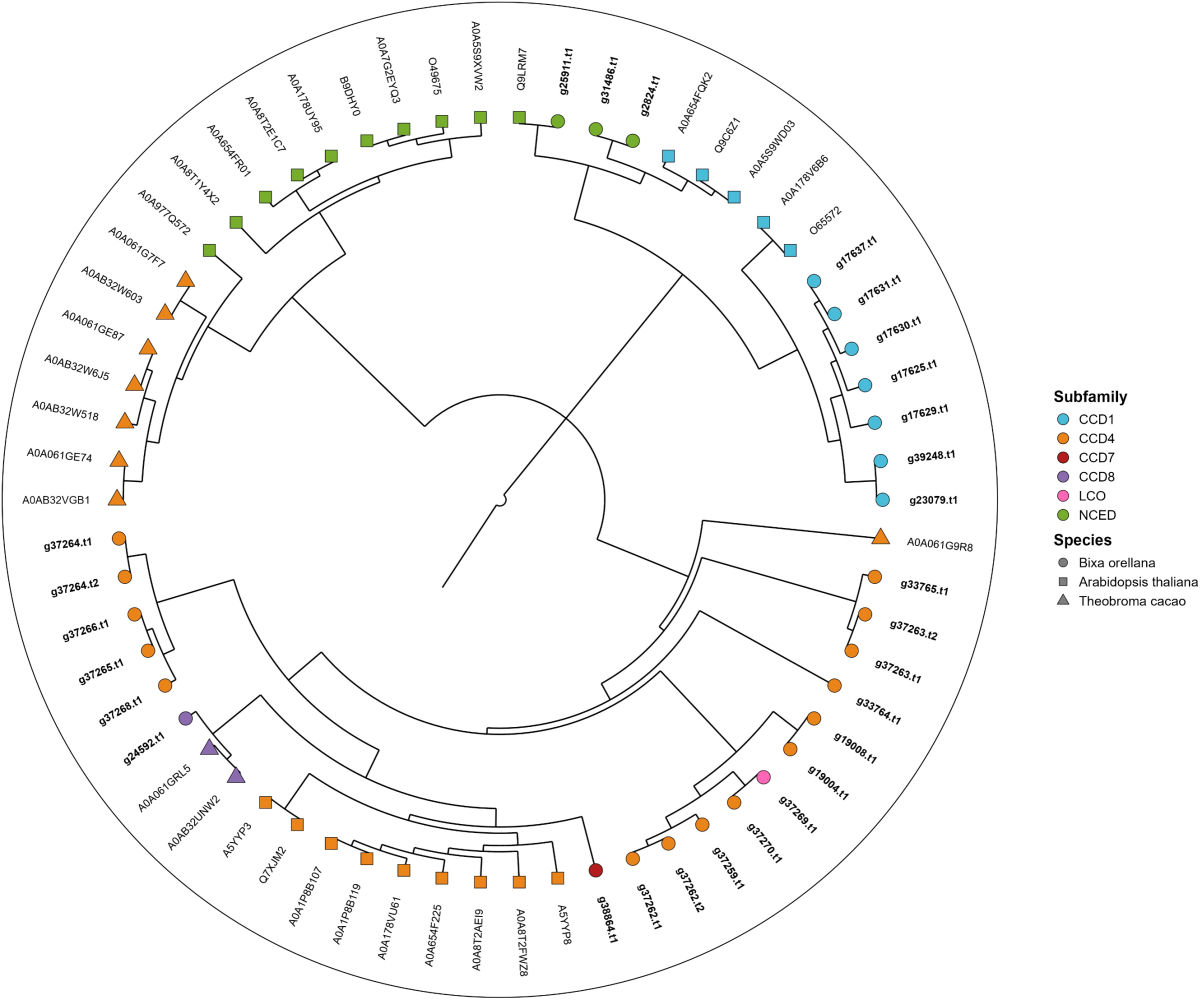
Circular maximum-likelihood phylogenetic cladogram of *B. orellana CCD* genes and their reference orthologs from *A. thaliana* and *T. cacao*. The tree was inferred using the WAG amino acid substitution model and rooted by midpoint. Branch lengths are not proportional to evolutionary distance (cladogram display). Tip symbols indicate species of origin: circles (*B. orellana* genomic sequences), squares (*A. thaliana*), and triangles (*T. cacao*). Fill colors denote subfamily classification: *CCD1* (blue), *CCD4* (orange), *CCD7* (dark red), *CCD8* (purple), *LCO* (pink), and *NCED* (green). Gene identifiers for *B. orellana* genomic sequences (g prefix) are shown in bold. Reference accessions with ambiguous phylogenetic placement or inconsistent annotations were excluded to maximize resolution of subfamily clades; specifically, nine *T. cacao* sequences initially annotated as *NCED4* were removed due to inconsistent annotations relative to their phylogenetic placement and high evolutionary distance from *B. orellana* sequences. The *CCD7* and *LCO* subfamilies are represented solely by *B. orellana* genomic sequences, as no reference ortholog with unambiguous phylogenetic placement was available in the curated dataset. The *CCD4* subfamily is additionally supported by one *T. cacao* sequence (A0A061G9R8, triangle) co-clustering with g37263.t1 and g37263.t2, and the *NCED* subfamily by ten *A. thaliana* orthologs (squares) co-clustering with g2824.t1, g31486.t1, and g25911.t1.

The complete 110-sequence alignment (*CCD*-related genes plus orthologous genes) was used to construct a maximum-likelihood phylogenetic tree visualized as a circular cladogram (**Figure 2**). The *CCD4* clade was the largest functional cluster, grouping *A. thaliana CCD4* (CCD4_ARATH), several *T. cacao CCD4* chloroplastic paralogs, and the full set of previously reported *B. orellana CCD4* isoforms. Notably, multiple novel genomic candidates also resolved within this clade (g17259.t1, g37262.t1, g37262.t2, g37264.t1, g37264.t2, g37265.t1, g37266.t1, g37268.t1, g17270.t1, g37263.t1, g37263.t2, g33764.t1, g33765.t1, g19004.t1, and g19008.t1), consistent with the known expansion of the *CCD4* family in *B. orellana* and their likely role in the oxidative cleavage of lycopene and β-carotene to generate bixin and norbixin, precursors. Specifically, the g37269.t1 accession shows a high level of separation from the *CCD4* clade, forming an isolated branch consistent with its assignment as a Lycopene Cleavage Oxygenase (*LCO*). The *CCD7* clade was resolved as a distinct monophyletic group, with the *B. orellana* sequence g38864.t1 placed within this group alongside *T. cacao CCD7* orthologs, suggesting a conserved role in strigolactone biosynthesis. The *CCD8* clade was resolved with *A. thaliana MAX4*/*CCD8* (CCD8_ARATH) and *T. cacao CCD8* orthologs. The *B. orellana* sequence g24592.t1 was placed within this group, suggesting a conserved role in the downstream conversion of carlactone intermediates in the strigolactone pathway. The *NCED* clade encompassed the well-characterized *A. thaliana* 9-cis-epoxycarotenoid dioxygenases (*NCED3*, *NCED5*, *NCED6*, *NCED9*) and several *T. cacao NCED4* sequences. Genes g2824.t1, g31486.t1, and g25911.t1 were grouped within the *NCED* clade, supported by ortholog identities of 48.63%, 49.54%, and 43.21% to characterized *A. thaliana NCED* sequences, respectively, which were consistently 2–2.5 times higher than their identities to any *CCD4* reference sequence. Considering the information from the preliminary circular dendrogram (**Supplementary Figure 2**) and the best-identified ortholog (**Supplementary Table 3**) for each *CCD*-related accession, a phylogenetic inference was conducted using an MRCA algorithm. In total, 28 accessions were included, as those showing ortholog identities <30% were discarded (g24585.t1: 18.35%; g24578.t1: 20.69%; g24581.t1: 22.54%; g24580.t1: 23.07%; g37989.t1: 28.47%). Additionally, g24582.t1 was excluded due to its extremely low identity to all characterized reference sequences from *A. thaliana* and *T. cacao* (maximum identity 30.86% to a divergent *CCD1-like* sequence from *T. cacao*), with no reliable ortholog assignment possible. For comparison, confirmed *CCD1* members of *B. orellana* showed ortholog identities ranging from 45.47% to 53.71% (mean 50.84%) against characterized *A. thaliana CCD1* orthologs, placing g24582.t1 more than 14 percentage points below the lowest confirmed *CCD1* member (*CCD1_copy5*, 45.47%), further supporting its exclusion. The resulting curated alignment of 28 accessions, confirmed as containing representatives of all major *CCD* subfamilies, was used to construct the final maximum-likelihood cladogram shown in **Figure 2**, which incorporates reference orthologs from *A. thaliana* and *T. cacao* to provide cross-species subfamily support.

The resulting tree revealed two primary well-supported clades, reflecting a clear subfamily partitioning among the 28 retained accessions. The tip symbols in the tree further discriminate the species of origin: circles represent *B. orellana* genomic sequences, squares represent *A. thaliana* orthologs, and triangles represent *T. cacao* orthologs. Reference accessions with ambiguous phylogenetic placement or inconsistent annotations were excluded from the final visualization to maximize clarity: AT_A0A7G2E155, TC_A0A061GZL5, AT_A0A654FB17, TC_A0A061DUB2, TC_A0A061DUG4, TC_A0AB32WEX7, AT_A0A178VEZ7, AT_Q8VY26, AT_A0A1P8B8T4, AT_Q9LRR7, AT_Q9M9F5, and AT_Q9FGC7 were removed as they did not provide unambiguous subfamily support. Additionally, nine *T. cacao* sequences initially annotated as *NCED4* (TC_A0A061GTU1, TC_A0A061GTU5, TC_A0A061GVR0, TC_A0AB32URQ8, TC_A0AB32UZS0, TC_A0AB32WZG6, TC_A0A061GAZ2, TC_A0A061FAU8, and TC_A0A061GRK8) were excluded due to inconsistent annotations relative to their phylogenetic placement and high evolutionary distance from *B. orellana* sequences. The *CCD7* (g38864.t1) and *LCO* (g37269.t1) subfamilies are represented solely by *B. orellana* genomic sequences, as no reference ortholog with unambiguous placement was available in the dataset. The first and largest clade was exclusively composed of sequences annotated as *CCD4* orthologs (shown in orange), comprising 15 accessions: g19004.t1, g19008.t1, g37259.t1, g37262.t1, g37262.t2, g33765.t1, g37270.t1, g37265.t1, g37266.t1, g37268.t1, g37264.t1, g37264.t2, g33764.t1, g37263.t1, and g37263.t2. The internal branching pattern within this group indicated a relatively recent diversification with short internal branches, suggesting functional conservation among these paralogs or recent gene duplication events within this subfamily. This *CCD4* clade is supported by co-clustering with *A. thaliana CCD4* orthologs (squares; including Q8VY26, A0A1P8B8T4, and related isoforms) and multiple *T. cacao CCD4* sequences (triangles; including A0AB32W603, A0A061GE87, A0AB32VGB1, among others), confirming the subfamily assignment through cross-species ortholog support. The second major clade showed greater subfamily diversity and encompassed the remaining accessions. Within this clade, a subclade of *CCD1*-annotated sequences (blue) was recovered with high internal resolution, grouping g17631.t1, g17637.t1, g17630.t1, g17625.t1, g17629.t1, g39248.t1, and g23079.t1. This subclade is anchored by *A. thaliana CCD1* orthologs (squares; O65572 and A0A178V6B6), providing cross-species support for the *CCD1* classification of these *B. orellana* genes. Notably, two *NCED*-assigned accessions (green), g25911.t1, g31486.t1, and g2824.t1, formed a distinct and well-separated lineage sister to the *CCD1* subclade, consistent with the known evolutionary relationship between *NCED* and *CCD1* enzymes, as both cleave carotenoid substrates at similar positions. The remaining accessions, g38864.t1 (*CCD7*, shown in dark red), g24592.t1 (*CCD8*, shown in purple), occupied isolated phylogenetic positions as long-branch taxa within this second clade, consistent with their expected functional divergence from the *CCD1*/*NCED* lineage. g37269.t1 (*LCO*, shown in pink) also formed an isolated long branch, reflecting its early divergence from other *CCD* subfamilies. *B. orellana CCD7* placement (g38864.t1) forms an isolated long branch without close ortholog support from either reference species in the curated tree, consistent with the exclusion of ambiguously placed *CCD7* reference sequences. *CCD8* is supported by co-clustering with *T. cacao* A0A061GRL5 and A0AB32UNW2 (triangles), confirming the subfamily assignment through cross-species ortholog support. The *NCED* subclade is corroborated by multiple *A. thaliana NCED* orthologs (squares; Q9LRM7, A0A5S9XVW2, A0A654FR01, A0A977Q572, A0A178UY95, A0A8T1Y4X2, A0A8T2E1C7, O49675, B9DHY0, and A0A7G2EYQ3), all well-characterized members of the ABA biosynthesis pathway. The *LCO* accession g37269.t1 remains without close ortholog support from either reference species in the curated tree, consistent with its classification as a lineage-specific lycopene cleavage oxygenase. Altogether, the topology of the maximum-likelihood tree supported the subfamilial assignments derived from the ortholog search and confirmed that the 28 curated accessions represent the major functional diversity of the *CCD* gene family in the studied organism. A final analysis of these 28 selected genes using the WoLF PSORT platform revealed differences in subcellular localization predictions: most *BoCCD1* and *BoCCD4* proteins were predicted to be primarily cytoplasmic. At the same time, the most phylogenetically distant *BoCCD4* isoforms (g25911.t1 and g31486.t1) showed a strong chloroplastic localization.

To further characterize the evolutionary dynamics of the *B. orellana CCD* family, pairwise synonymous (Ks) and non-synonymous (Ka) substitution rates were estimated from codon-level alignments for all 378 gene pairs among the 28 curated sequences (**Supplementary Table 4**). Mean pairwise Ks values ranged from 0.367 (g17637.t1, *CCD1*) to 1.449 (g23079.t1, *CCD1*), with the *CCD1* subfamily showing the lowest within-subfamily Ka/Ks ratio (mean 0.655), consistent with predominant purifying selection. Within-subfamily *CCD4* comparisons yielded a mean Ka/Ks of 1.296, suggesting relaxed selective constraint or episodic diversifying selection among the expanded *CCD4* paralogs. Notably, g33764.t1 and g37259.t1 showed the highest mean Ks values within the *CCD4* clade (1.244 and 1.265, respectively), indicating earlier divergence from the core *CCD4* members. Pairs involving highly divergent subfamilies (*CCD1* vs *NCED*, *NCED* vs *NCED*) returned undefined Ks values due to saturation of synonymous sites, a known limitation of the NG86 method for distantly related sequences. The final assignment of all 28 *B. orellana CCD* genes, including subfamily classification, subcellular localization predictions, ortholog identities, and mean pairwise Ks values, is summarized in **Table 2**.

**Table 2:** Identity and assigned function of the *CCD*-related genes.

Code	Sub family	Gene id	WoLF PSORT	Best ortholog (Species)	Ortholog function	Identity %	mean Ks	Phylogenetic placement (Supplementary Figure 2)
CCD4-1	CCD4	g19008.t1	ch:6, cy:4, mi:2, nu:1, ex:1	A0A9Y0ZEY2 (B. orellana)	CCD4-1	100	0.732	CCD4 clade; plastid-localized branch
CCD4-3	CCD4	g37270.t1	ch:6, cs:3, nu:1, cy:1, va:1, go:1	A0A9Y0ZEY5 (B. orellana)	CCD4-3	100	0.659	CCD4 clade; high bootstrap support
CCD4-4	CCD4	g37262.t1	ch:8, ex:2, nu:1, mi:1, va:1, er:1	A0A9Y0ZFW6 (B. orellana)	CCD4-4	100	0.542	Distinct CCD4 sub-clade; plastid-targeted
CCD4-2	CCD4	g37268.t1	cy:6, cs:4, ch:3, nu:1	A0A9Y0ZFT2 (B. orellana)	CCD4-2	92.42	0.695	Core CCD4–2 sub-clade
CCD4-2_copy1	CCD4	g37265.t1	cy:6, cs:4, ch:3, nu:1	A0A9Y0ZFT2 (B. orellana)	CCD4-2	89.28	0.733	CCD4–2 sub-clade; recent duplication
CCD4-2_copy2	CCD4	g37266.t1	nu:5.5, nu, pl:5, pl:3.5, ch:3, cy:2	A0A9Y0ZFT2 (B. orellana)	CCD4-2	75.51	0.678	CCD4–2 sub-clade; divergent copy
CCD4-2_copy3	CCD4	g37264.t1	cy:13, ex:1	A0A9Y0ZFT2 (B. orellana)	CCD4-2	70.12	0.452	Peripheral CCD4–2 sub-clade
CCD4-2_copy4	CCD4	g37264.t2	cy:7, ch:4, mi:2, pe:1	A0A9Y0ZFT2 (B. orellana)	Probable CCD4-2	38.41	0.714	Basal CCD4-2; long branch
CCD4-2_copy5	CCD4	g33764.t1	cy:12, ch:1, mi:1	A0A061GVR0 (T. cacao)	Probable CCD4-2	40.96	1.244	Basal CCD4; early divergence in Malvales
CCD4-4_copy1	CCD4	g37259.t1	ch:10, nu:1, cy:1, ex:1, er, va:1	A0A9Y0ZFB6 (B. orellana)	CCD4-4	42.92	1.265	CCD4–4 sub-clade; functional divergence
CCD4-4_copy2	CCD4	g37262.t2	cy:11, ch:1, nu:1, mi:1	A0A9Y0ZFV9 (B. orellana)	CCD4-4	100	0.906	CCD4-4; likely isoform or allelic variant
CCD4_chl	CCD4	g19004.t1	cy:5, ch:4, go:2, nu:1, ex:1, pe:1	A0A9Y0ZFZ3 (B. orellana)	CCD4-like	86.33	0.616	CCD4-like; divergent plastid-targeted paralog
CCD4_chl1	CCD4	g33765.t1	ch:5, cy:4, nu:2, mi:1, va:1, er:1	A0A9Y0ZFZ9 (B. orellana)	CCD4-like	50	0.81	Peripheral CCD4 clade; significant divergence
CCD4_chl2	CCD4	g37263.t1	ch:13, ex:1	A0A061G9R8 (T. cacao)	NCED4	41.19	0.452	CCD4/NCED boundary; proximity to NCED
CCD4_chl3	CCD4	g37263.t2	ch:12, nu:1, mi:1	A0A061G9R8 (T. cacao)	NCED4	41.19	0.704	CCD4/NCED interface; sister to CCD4_chl2
CCD1	CCD1	g17625.t1	cy:9, nu:2, pe:2, mi:1	O65572 (A. thaliana)	CCD1	53.22	0.594	Anchors CCD1 clade with AtCCD1
CCD1_copy1	CCD1	g17629.t1	cy:8, mi:3, nu:2, pe:1	O65572 (A. thaliana)	CCD1	53.51	0.913	CCD1 clade; recent duplication
CCD1_copy2	CCD1	g17630.t1	cy:5, nu:3, er:3, va:2, pl:1	O65572 (A. thaliana)	CCD1	50.81	0.68	CCD1 clade; ER/vacuolar signals
CCD1_copy3	CCD1	g17631.t1	pe:13, cy:1	O65572 (A. thaliana)	CCD1	53.71	0.615	CCD1 clade; peroxisomal targeting
CCD1_copy4	CCD1	g17637.t1	pe:13, cy:1	O65572 (A. thaliana)	CCD1	53.51	0.367	CCD1 clade; peroxisomal; functional redundancy
CCD1_copy5	CCD1	g23079.t1	pe:13, cy:1	O65572 (A. thaliana)	CCD1	45.47	1.449	Peripheral CCD1 clade; peroxisomal
CCD1_copy6	CCD1	g39248.t1	pe:14	A0A178V6B6 (A. thaliana)	CCD1	45.64	0.717	Basal CCD1; exclusive peroxisomal prediction
CCD7	CCD7	g38864.t1	ch:11, pe:3	A0A061DUB2 (T. cacao)	CCD7	58.25		Sole CCD7; distinct monophyletic group
CCD8	CCD8	g24592.t1	cy:5, ch:4, mi:2, nu:1, pl:1, cs:1	A0A061GRL5 (T. cacao)	CCD8	55.8	0.463	Single CCD8; strigolactone pathway
LCO	LCO	g37269.t1	ch:13, mi:1	F8SRI5 (B. orellana)	LCO	32.92	0.453	Isolated LCO; long branch; early divergence
NCED	NCED	g2824.t1	mi:7, ch:4, nu:1, cy:1, cs:1	A0A178VEZ7 (A. thaliana)	NCED	48.63	0.631	NCED clade; mitochondrial/plastid targeting
NCED_copy1	NCED	g31486.t1	ch:13, mi:1	A0A178VEZ7 (A. thaliana)	NCED	49.54	0.925	NCED clade; functional redundancy in ABA
NCED6	NCED6	g25911.t1	ch:9, mi:2, cy:1, er:1, pe:1	Q9LRM7 (A. thaliana)	NCED6	43.21	0.766	Distinct NCED6 sub-clade; AtNCED6 ortholog

### 3.5 Comparative gene structure analysis reveals distinct architectural patterns among *B. orellana CCD* family members

**Figure 3:**
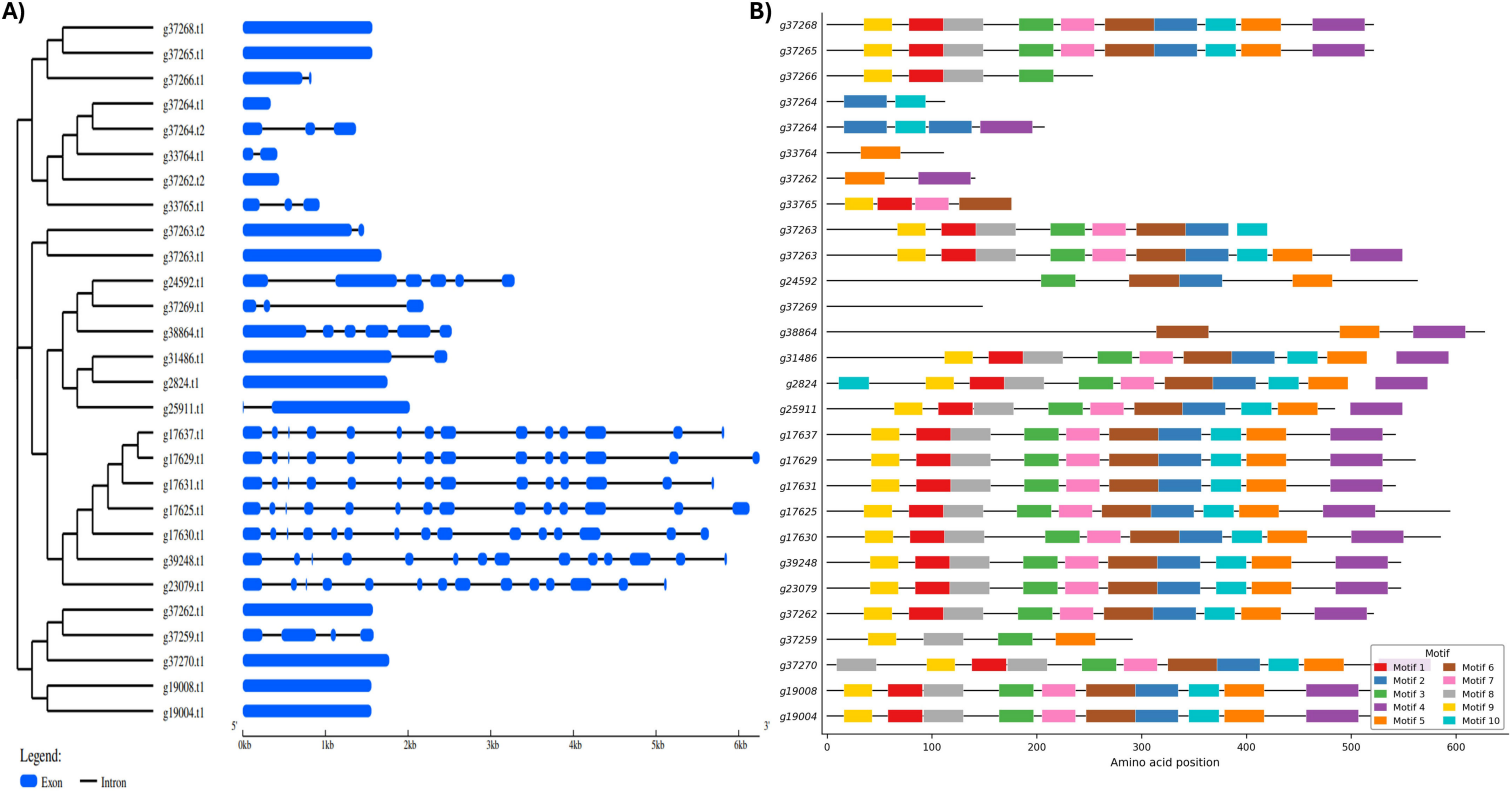
Comparative gene structure and conserved motif distribution of the *B. orellana CCD* gene family. **(A)** Phylogenetically ordered gene structure visualization showing exon–intron organization of 28 *CCD* genes. The dendrogram (left) depicts evolutionary relationships inferred by maximum-likelihood. Blue boxes represent exons; black lines denote introns. **(B)** MEME-based conserved protein motif distribution across the 28 *CCD* sequences, displayed in the same phylogenetic order as panel **(A)** Each horizontal bar represents a protein sequence scaled proportionally to its length in amino acids. Colored rectangles indicate the position and identity of each of the 10 motifs detected (Motif 1–10; color-coded as shown in the legend). Sequences with no detected motifs (e.g., *LCO*/g37269) are shown as backbone lines only.

The genomic architecture of *CCD* genes in *B. orellana* exhibited remarkable structural diversity, as illustrated by the phylogenetic gene structure visualization (**Figure 3**). Gene structures were organized according to their evolutionary relationships inferred from the maximum-likelihood dendrogram. Two major structural groups were distinguished by their complexity of exon–intron organization. The upper clade of the dendrogram encompassed gene g37268.t1, g37265.t1, and g37266.t1, which predominantly displayed simple architectures characterized by few exons and large continuous coding regions. Within a related subclade, genes such as g37263.t2, g37263.t1, g2824.t1, g25911.t1, g37262.t2, g19008.t1, and g19004.t1 exhibited essentially intronless or single-exon structures with minimal intron content. Other members, including g24592.t1, g37269.t1, g38864.t1, g31486.t1, g33765.t1, g37259.t1, g37262.t1, and g37270.t1, displayed two- to three-exon organizations with variable intron spacing. Among these, mean pairwise Ks values varied considerably: g24592.t1 (Ks = 0.463) and g37269.t1 (Ks = 0.453) showed the lowest synonymous divergence within this group, while g37259.t1 (Ks = 1.265) and g31486.t1 (Ks = 0.925) exhibited the highest, reflecting greater evolutionary distance from other *B. orellana CCD* members. In contrast, the complex-architecture clade comprises gene g39248.t1, g23079.t1, g17637.t1, g17631.t1, g17629.t1, g17625.t1, and g17630.t1 were characterized by highly fragmented gene structures, with numerous short exons (10–15+) interspersed with multiple introns spanning the entire transcript, extending up to 6 kb. This group showed a consistent multi-exon pattern, with regular spacing; mean pairwise Ks values ranged from 0.367 (g17637.t1) to 1.449 (g23079.t1), with g17637.t1 and g17625.t1 showing the lowest divergence (Ks = 0.367 and 0.594, respectively), consistent with stronger selective constraint on these core *CCD1* members. A third group comprising g33764.t1, g37264.t1, and g37264.t2 clustered separately in the dendrogram; g37264.t2 displayed a three-exon structure with two prominent introns and carried the highest observed mean Ks value among the three (Ks = 0.714), while g37264.t1 (Ks = 0.452) presented a nearly intronless architecture. Notably, g33764.t1 showed the highest Ks value in this group (Ks = 1.244), suggesting earlier divergence from the core *CCD4* members. Complete pairwise Ka, Ks, and Ka/Ks values for all gene comparisons are provided in **Supplementary Table 4**. To complement structural comparisons, a GLAM2 motif analysis was performed on CCD protein sequences to identify conserved amino acid patterns (**Supplementary Figure 3**). The alignment revealed a highly conserved motif of approximately 48–50 aa present in 21 CCD proteins, corresponding to a region likely important for carotenoid cleavage activity. To further characterize the functional conservation of *B. orellana* CCD proteins, a MEME-based motif analysis identified 10 conserved sequence motifs distributed across the 28 curated proteins (**Figure 3B**).

Sequences with full-length architectures, corresponding to the *CCD1* and *CCD4* subfamilies (e.g., g17625.t1, g17629.t1, g17630.t1, g17631.t1, g17637.t1, g37268.t1, g37265.t1, g19008.t1, g19004.t1), consistently harbored the complete complement of all 10 motifs in a conserved linear arrangement, with motifs 1, 3, 6, and 4 corresponding to the iron-binding, RPE65-type, and C-terminal dioxygenase signature regions. In contrast, structurally simpler or shorter sequences showed partial motif sets: g37266 and g33765 retained only 4 motifs, while the truncated sequences g33764, g37264, and g37269 (*LCO*) harbored 1, 2, and 0 motifs, respectively, consistent with their divergent or incomplete protein structures. The *NCED* members (g31486, g2824, g25911) and *CCD8* (g24592) displayed 10 and 4 motifs respectively, with overall arrangements comparable to the *CCD1*/*CCD4* group but with subfamily-specific variations in motifs 7 and 9, consistent with their distinct substrate specificities. The complete conservation of motifs 1, 3, and 6 across all full-length members underscores the evolutionary constraint on the catalytic core of the *CCD*/*RPE65* superfamily in *B. orellana*.

The GLAM2 algorithm detected semi-conserved sequence motifs with variable-length gaps, indicating structurally flexible yet functionally constrained regions. Three sequence logos were generated at different GLAM2 scores (2308.17, 2235.49, and 2160.64), consistently highlighting residues with high information content, notably tryptophan, phenylalanine, aspartate, and glycine, suggestive of their involvement in maintaining the catalytic or substrate-binding core of CCD proteins, as further reflected by the recurring FDGDG-type pattern visible across the aligned sequences. This clade corresponded to the structurally complex genes under strong purifying selection, suggesting functional conservation of the carotenoid cleavage catalytic core. In contrast, *CCD-like* sequences not recovered by the motif search may represent divergent or non-canonical *CCD* members. The high conservation within this motif supports the hypothesis that despite the exon–intron diversity observed at the genomic level, CCD proteins retain a conserved functional domain essential for enzymatic activity.

### 3.6 Promoter analysis and identification of cis-regulatory elements

**Figure 4:**
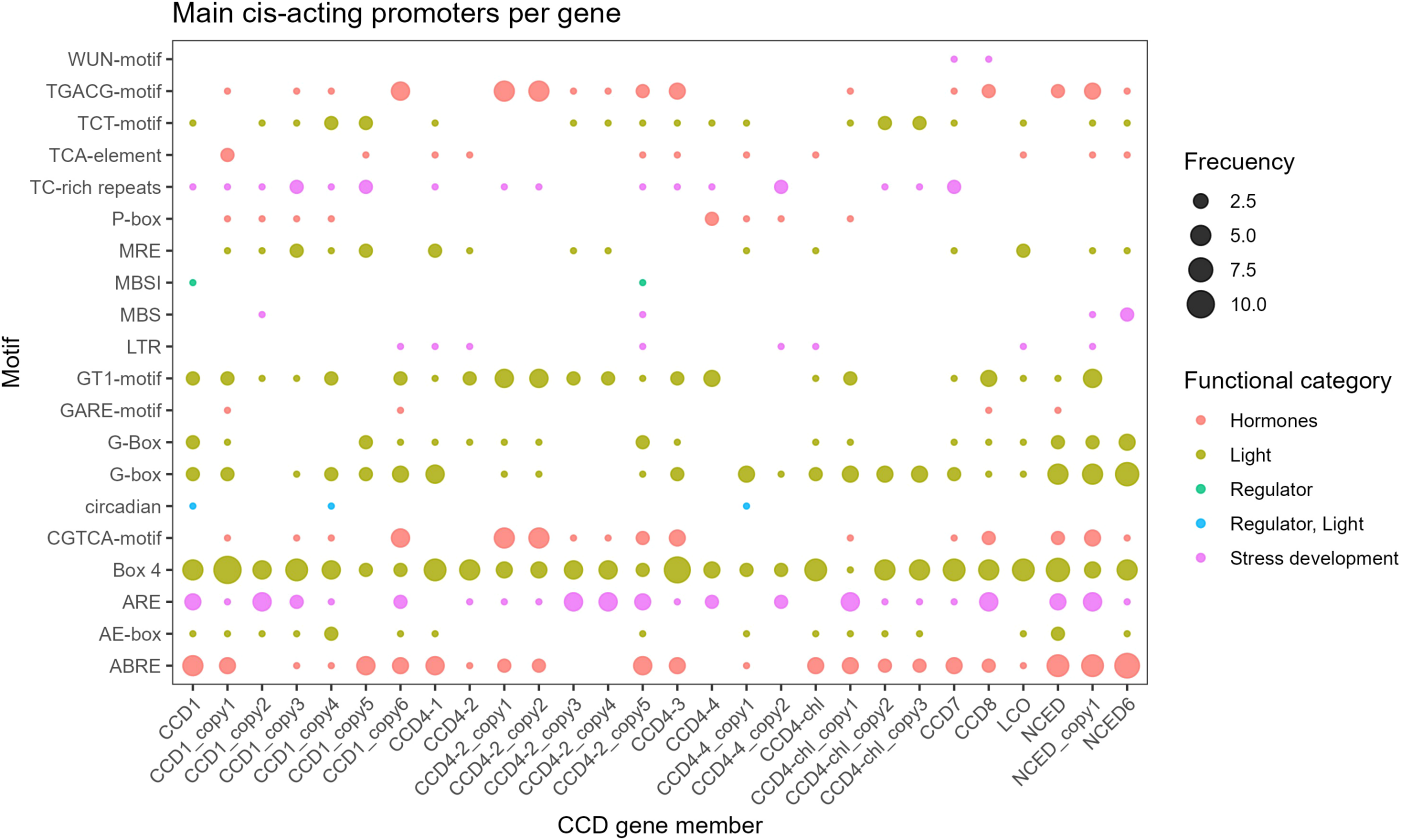
Main cis-acting promoters per *CCD* gene. The y-axis lists individual motif names, and the x-axis represents different *CCD* genes. Dot color indicates the functional category of the motif, and dot size corresponds to its frequency in each gene’s promoter.

To elucidate the transcriptional regulation of *CCD* gene members in *B. orellana*, we conducted an *in silico* analysis of their promoter regions. The 2,000 bp upstream region of all *CCD* genes was analyzed using the PlantCARE database to classify cis-acting regulatory elements. Our analysis revealed a diverse predicted regulatory potential landscape, with 556 high-interest cis-elements identified across the promoters of the *CCD* genes (**Supplementary Table 6**). These elements were systematically categorized into four major functional groups: Light responsiveness, Hormone responsiveness, Stress development, and general transcriptional Regulators (including circadian control). The distribution and abundance of these elements are visualized with a dot plot overview in **Figure 4**, demonstrating that high-interest motifs are ubiquitous among *CCD* promoters, though their composition varies significantly. Light-responsive elements were the most abundant and widely distributed category, with G-box, Box 4, GT1-motif, TCT-motif, and AE-box being present in nearly all *CCD* genes. This broad prevalence is consistent with a predicted involvement of light-responsive regulatory mechanisms acting on members of the *CCD* gene family. Hormone-responsive elements were also highly prevalent, with motifs associated with abscisic acid (ABRE), MeJA (CGTCA-motif, TGACG-motif), gibberellin (GARE-motif, P-box), and salicylic acid (TCA-element) being frequently identified. Furthermore, promoters were enriched with stress-responsive elements, including ARE (anaerobic induction), TC-rich repeats (defense and stress), LTR (low-temperature), MBS (drought-inducibility), and WUN-motif (wound-responsiveness), consistent with the potential responsiveness of *CCD* gene expression to environmental stress-related signals. Additionally, regulatory elements with dual roles in light sensing and circadian control, including MBSI and the circadian element, were identified in a subset of genes, as were metal-response elements (MREs), suggesting additional layers of predicted transcriptional regulatory potential within this gene family. It should be noted that all identifications are based on sequence similarity to known motifs in the PlantCARE database and represent inferred regulatory potential; experimental validation would be required to confirm their functional activity.

**Figure 5:**
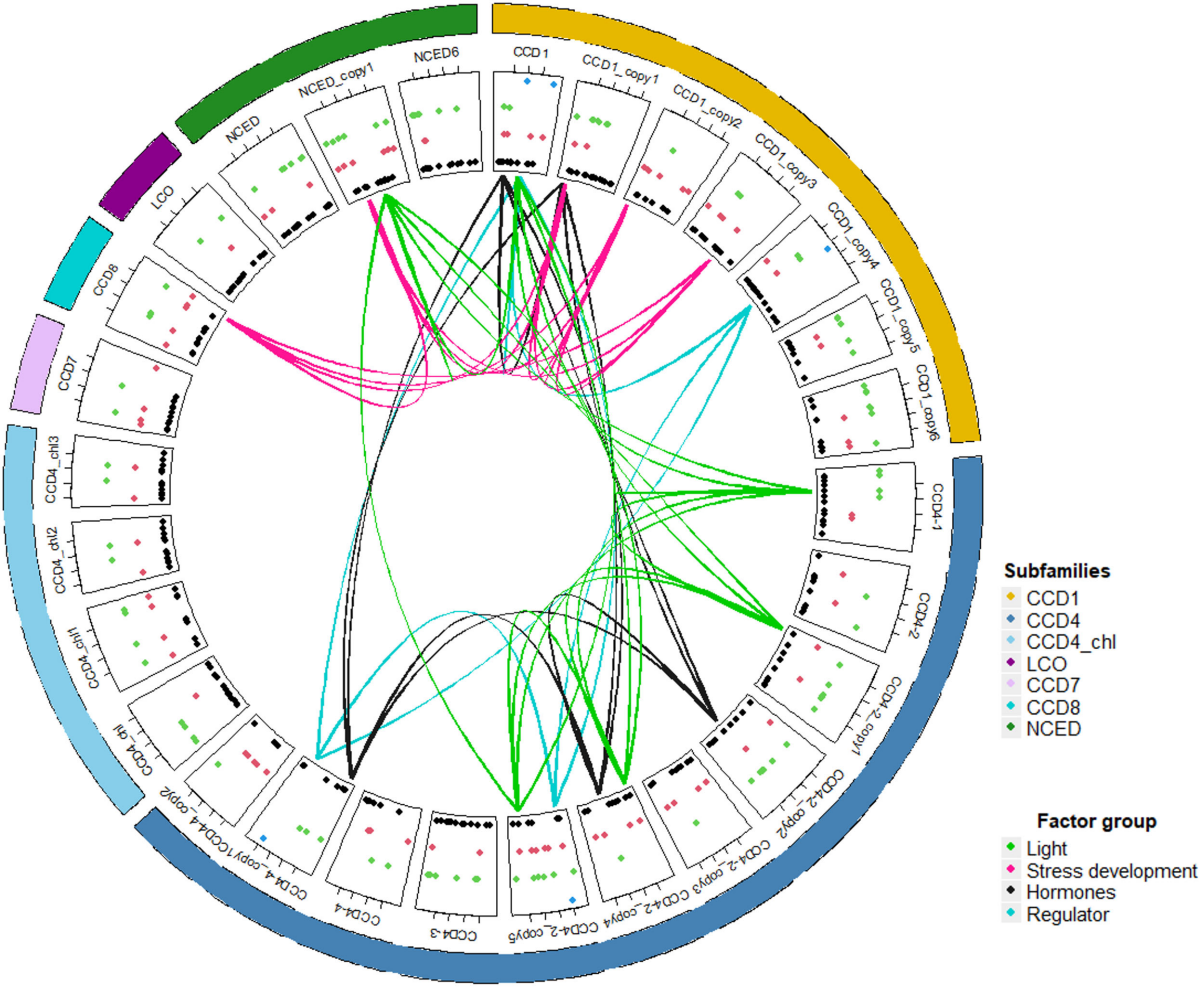
Circos-style plot shows the distribution and shared motif profile similarity of cis-elements across *B. orellana CCD* genes. Theouter track displays *CCD* genes color-coded by subfamily: The outer track displays *CCD* genes color-coded by subfamily: *CCD1* (gold), *CCD4* (blue), *CCD4_chl* (cyan), *LCO* (purple), *CCD7* (lavender), *CCD8* (teal), and *NCED* (dark green). The inner scatter points represent the position of individual cis-elements within the 2,000 bp promoter region, colored by functional category: Light (green), Stress development (pink), Hormones (black), and Regulator (cyan). Curved links connect genes that share a high abundance of cis-elements within the same functional category, with link color corresponding to the shared factor group, indicating similar promoter element composition across and within subfamilies.

An integrative circular plot (**Figure 5**) provides a spatial and relational view of these regulatory features. The arrangement of genes by subfamily reveals that members of the *CCD1* and *CCD4–2* subfamilies exhibit particularly dense and diverse arrays of cis-elements. The positional mapping along the promoter length shows no strong bias, with light, hormone, stress development, and regulator elements distributed throughout the 2,000 bp region across all subfamilies. The curved links connecting genes highlight shared regulatory element profiles among *CCD* promoters, revealing that genes from different subfamilies can share similar regulatory potentials. Hormone-associated links (black) are the most numerous and broadly distributed, interconnecting *CCD1*, *CCD4-2*, *NCED*, and *CCD4_chl* members, suggesting widespread coordinated responses to phytohormone signals. Light-responsive links (green) are also abundant, particularly connecting *CCD1* and *CCD4–2* genes, while Stress development (pink) and Regulator (cyan) links delineate more selective co-regulatory relationships among specific gene pairs. These results suggest that the promoter architecture of the *B. orellana CCD* gene family is characterized by a rich diversity of cis-regulatory elements. The prevalence of light- and hormone-related motifs strongly suggests that the expression of these genes is under complex transcriptional control, integrating multiple internal and external signals to regulate apocarotenoid metabolism finely. It is important to note that cis-regulatory element identification reflects regulatory potential inferred from sequence features and does not constitute direct evidence of transcriptional activity or regulatory causality.

### 3.7 Chromosomal distribution and genomic localization of *CCD* genes

**Figure 6:**
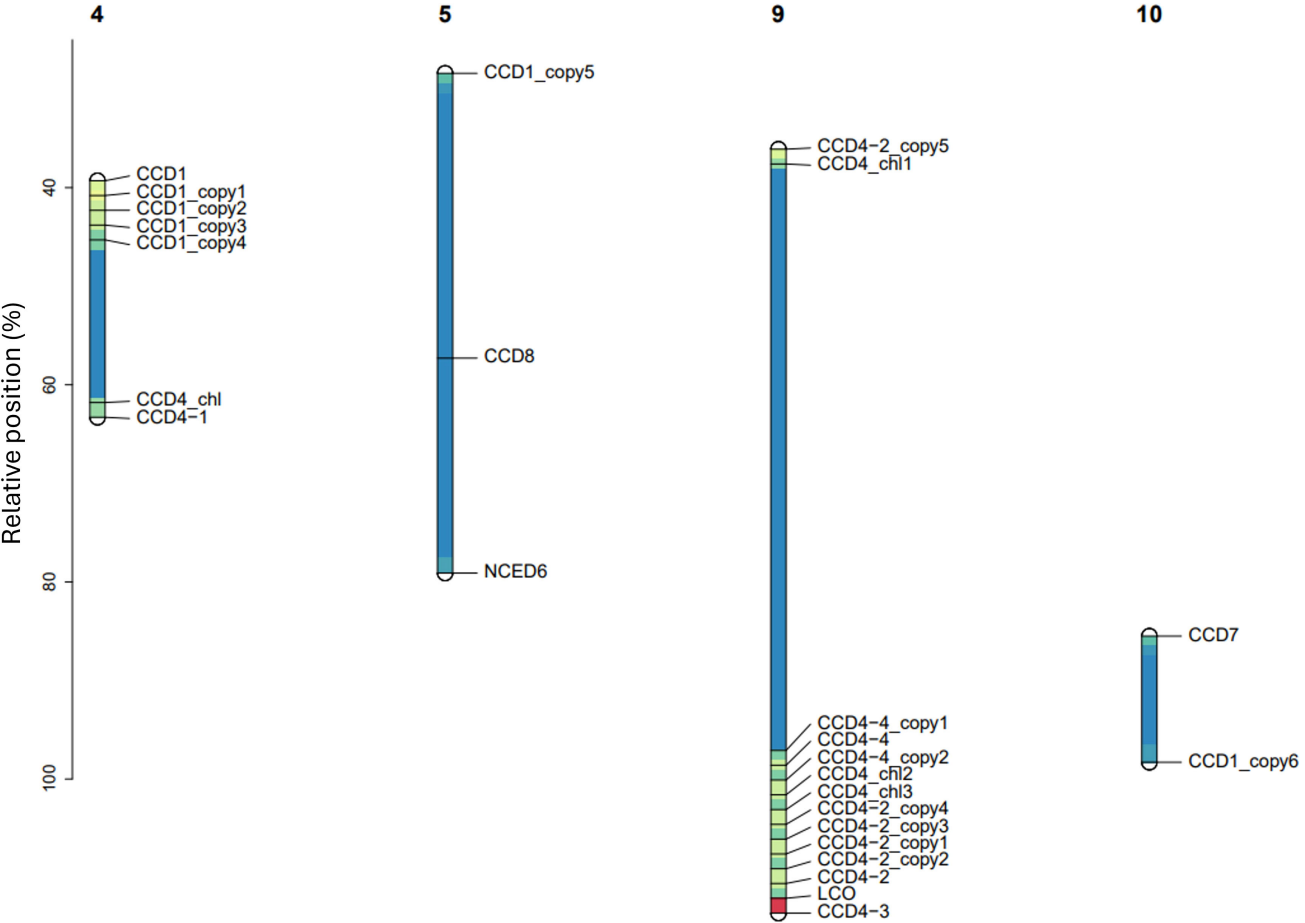
Chromosomal distribution and genomic location of *B. orellana CCD* genes. The linkage map shows the location of the *CCD* genes across the scaffolds with the highest gene density (4, 5, 9, and 10). Gene positions are expressed as relative position (%) within each scaffold, normalized independently to allow uniform visual comparison regardless of scaffold length (scaffold absolute lengths range from ~12.9 Mb to ~40.8 Mb). Genes are labeled with their subfamily names and positioned to the right of each scaffold bar. The color gradient within each scaffold represents the local density of *CCD* genes, with warm colors (red, orange) indicating regions of high gene clustering and cool colors (blue, green) indicating regions of dispersed distribution. The tandemly duplicated *CCD1* genes at the proximal end of scaffold 4 and, most prominently, the large *CCD4* cluster at the distal end of scaffold 9 stand out as the principal genomic hotspots of the *CCD* gene family. Scaffold absolute lengths: scaffold 4 = 40.8 Mb; scaffold 5 = 35.2 Mb; scaffold 9 = 29.7 Mb; scaffold 10 = 12.9 Mb. Relative positions were calculated as (gene start/scaffold length) × 100.

To understand the genomic organization of the carotenoid cleavage dioxygenase (*CCD*) family in *B. orellana*, we mapped all identified *CCD* genes onto their respective chromosomal scaffolds. This analysis aimed to delineate their distribution patterns and identify potential genomic clusters indicative of recent duplication events. The *CCD* genes were found to be distributed across six scaffolds (ptg000001l, ptg000004l, ptg000005l, ptg000006l, ptg000009l, and ptg000010l), with a notably non-random organization. As illustrated in the linkage map-style visualization (**Figure 6**), scaffolds 4, 5, 9, and 10 contained significant densities of *CCD* genes and are displayed in the figure. Scaffolds 1 and 6, each carrying a single *CCD* gene (*NCED* and *NCED_copy1*, respectively), were excluded from figure as they do not contribute to the visualization of clustering patterns.

Gene positions expressed as relative coordinates (% of total scaffold length) to allow uniform visual comparison across scaffolds of differing absolute sizes (ranging from ~12.9 Mb to ~40.8 Mb). In regions of high gene density, particularly at the proximal end of scaffold 4 and the distal end of scaffold 9, a minimum positional offset was applied between adjacent labels for readability purposes; this offset does not alter the underlying genomic coordinates, which are provided in **Supplementary Table 1**. A striking pattern of gene clustering was observed on two scaffolds. Scaffold 4 harbors a compact cluster of *CCD1* subfamily members at its proximal end, comprising *CCD1*, *CCD1_copy1*, *CCD1_copy2*, *CCD1_copy3*, and *CCD1_copy4*, along with *CCD4_chl* and *CCD4–1* at a more distal position. The tight physical proximity of these phylogenetically related genes provides strong evidence for tandem duplication events as a key mechanism driving the expansion of the *CCD1* subfamily in *B. orellana*. Scaffold 1, by contrast, carries a single gene, *NCED*, located at an intermediate position, indicating that this scaffold contributes to *CCD* family diversity without local gene clustering.

Further analysis using a sliding-window approach to calculate local gene density revealed distinct genomic hotspots for *CCD* genes, visualized as a color gradient from cool (low density) to warm (high density). The most intense warm colors (red/orange) are concentrated at the distal end of scaffold 9, where a remarkably dense cluster of *CCD4* subfamily members accumulates, including *CCD4-4*, *CCD4-4_copy1*, *CCD4-4_copy2*, *CCD4_chl2*, *CCD4_chl3*, *CCD4–2* and its multiple copies (*CCD4-2_copy1* through *CCD4-2_copy4*), *CCD4-3*, and *LCO*. This region constitutes the principal genomic hotspot of the entire *CCD* family. Scaffold 9 also carries *CCD4-2_copy5 and CCD4_chl1* near its proximal end, further underscoring its role as the primary locus of *CCD4* diversification. In contrast, scaffolds 5 and 10 showed a more dispersed distribution, harboring singletons such as *CCD1_copy5*, *CCD8*, and *NCED6* on scaffold 5, and *CCD7* and *CCD1_copy6* on scaffold 10. This mixed pattern of densely clustered and scattered localization suggests that the *CCD* family has evolved through a combination of tandem and segmental or whole-genome duplications. Taken together, these results indicate that the genomic landscape of the *CCD* family is characterized by significant clustering on two main scaffolds: scaffold 4, which concentrates the *CCD1* tandem array, and scaffold 9, which harbors the largest and most diverse *CCD4* cluster in the genome. This organization implies that tandem duplication has been a major evolutionary force, potentially facilitating the functional diversification of specific *CCD* subfamilies critical for apocarotenoid biosynthesis in *B. orellana*.

#### 3.7.1 Synteny conservation of *CCD* genes across reference species

To evaluate the conservation of genomic neighborhood across species, synteny analysis was performed for all six *CCD* subfamilies, comparing each *B. orellana* locus with its best-matched ortholog in *A. thaliana* and *T. cacao* (**Supplementary Figure 5**). The *CCD1* subfamily displayed a compact tandem array on scaffold 4, with *CCD1* through *CCD1_copy4* sharing a conserved genomic neighborhood. Additionally, *CCD4_chl* (g19004.t1) and *CCD4-1* (g19008.t1) are also located on scaffold 4 at a more distal position (~60% relative position), physically separated from the *CCD1* tandem array. Comparison with *A. thaliana AtCCD*1 (53.7% identity) and *T. cacao TcCCD1-like* (30.9%) showed partial conservation of flanking gene order, consistent with a shared ancestral locus that has undergone lineage-specific expansion in *B. orellana*. The *CCD4* subfamily, the most structurally complex, showed a dense cluster predominantly at the distal end of scaffold 9, with *CCD4-2*, *CCD4-2_copy1* through copy4, *CCD4_chl2*, *CCD4_chl3*, *CCD4-3*, *CCD4-4*, *CCD4-4_copy1*, *CCD4-4_copy2*, and *LCO* concentrated at the distal end (~90–100%), while *CCD4-2_copy5* and *CCD4_chl1* are located at the proximal region (~42%) of the same scaffold. Synteny with *T. cacao TcNCED4* (41.2% identity) and *A. thaliana AtNCED4* (31.5%) revealed partial conservation of flanking regions, though the degree of synteny was reduced compared to *CCD1*, reflecting the greater divergence of the *CCD4* clade. The *CCD7* and *CCD8* subfamilies each corresponded to a single copy gene (*g38864* and *g24592*, respectively), and both showed detectable flanking gene conservation with their *T. cacao* and *A. thaliana* orthologs (*TcCCD7*, 58.2%; *AtCCD7*, 42.4%; *TcCCD8*, 55.8%; *AtCCD8*, 41.0%), consistent with their conserved roles in strigolactone biosynthesis. The *LCO* member (g37269) is physically embedded within the *CCD4* cluster on scaffold 9, co-localizing with *CCD4-2_copy2* and *CCD4-3*, and displayed no detectable flanking synteny with reference species, suggesting lineage-specific repositioning or origin. The *NCED* subfamily members (g2824, g31486, g25911) are distributed across three different scaffolds, and synteny with *A. thaliana AtNCED3* (49.5% identity) and *T. cacao TcNCED4* (19.6%) was only partially conserved, as expected given the moderate sequence identity and the role of structural rearrangements in *NCED* diversification. Altogether, these results confirm that while the enzymatic core of *CCD* genes is broadly conserved across the Malvales and Brassicales, the genomic context of *B. orellana CCD* loci has been substantially reorganized, primarily through tandem duplication events concentrated on scaffolds 4 and 9.

#### 3.7.2 Formal paralog pair analysis of tandem duplication

To formally evaluate tandem duplication as the primary mechanism driving *B. orellana CCD* family expansion, a paralog pair analysis was conducted integrating genomic distance and synonymous substitution rates for all 116 same-scaffold gene pairs among the 28 curated *CCD* genes. Applying three simultaneous criteria; same scaffold, genomic distance < 200 kb, and mean pairwise Ks < 1.0, identified 39 tandem duplication candidate pairs distributed across two principal genomic regions (**Supplementary Table 5**; **Supplementary Figure 6**). On scaffold 4, 10 *CCD1* paralog pairs were identified within the tandem array spanning 11.3–78.8 kb, with a mean pairwise Ks of 0.162, indicating relatively recent duplication events under strong selective constraint. An additional *CCD4* paralog pair (*CCD4_chl*/*CCD4-1*; distance = 12.1 kb, Ks = 0.094) was also identified on scaffold 4. On scaffold 9, 28 *CCD4* paralog pairs were identified within the distal cluster spanning 0.1–47.7 kb, with a mean pairwise Ks of 0.403, reflecting older or more divergent duplication events consistent with the greater functional diversification observed within this subfamily. Three paralog pairs showed Ks = 0 (g37262.t1/t2, g37263.t1/t2, and g37265/g37266), indicating near-identical or very recently duplicated sequences. Notably, five additional *CCD4* pairs within 200 kb showed Ks ≥ 1.0, suggesting synonymous site saturation in the more divergent members of the scaffold 9 cluster. Together, these results provide formal quantitative support for tandem duplication as the primary evolutionary force driving *CCD* family expansion in *B. orellana*, concentrated in two distinct genomic hotspots on scaffolds 4 and 9.

### 3.8 Transcriptomic expression profiling of *CCD* genes

**Figure 7:**
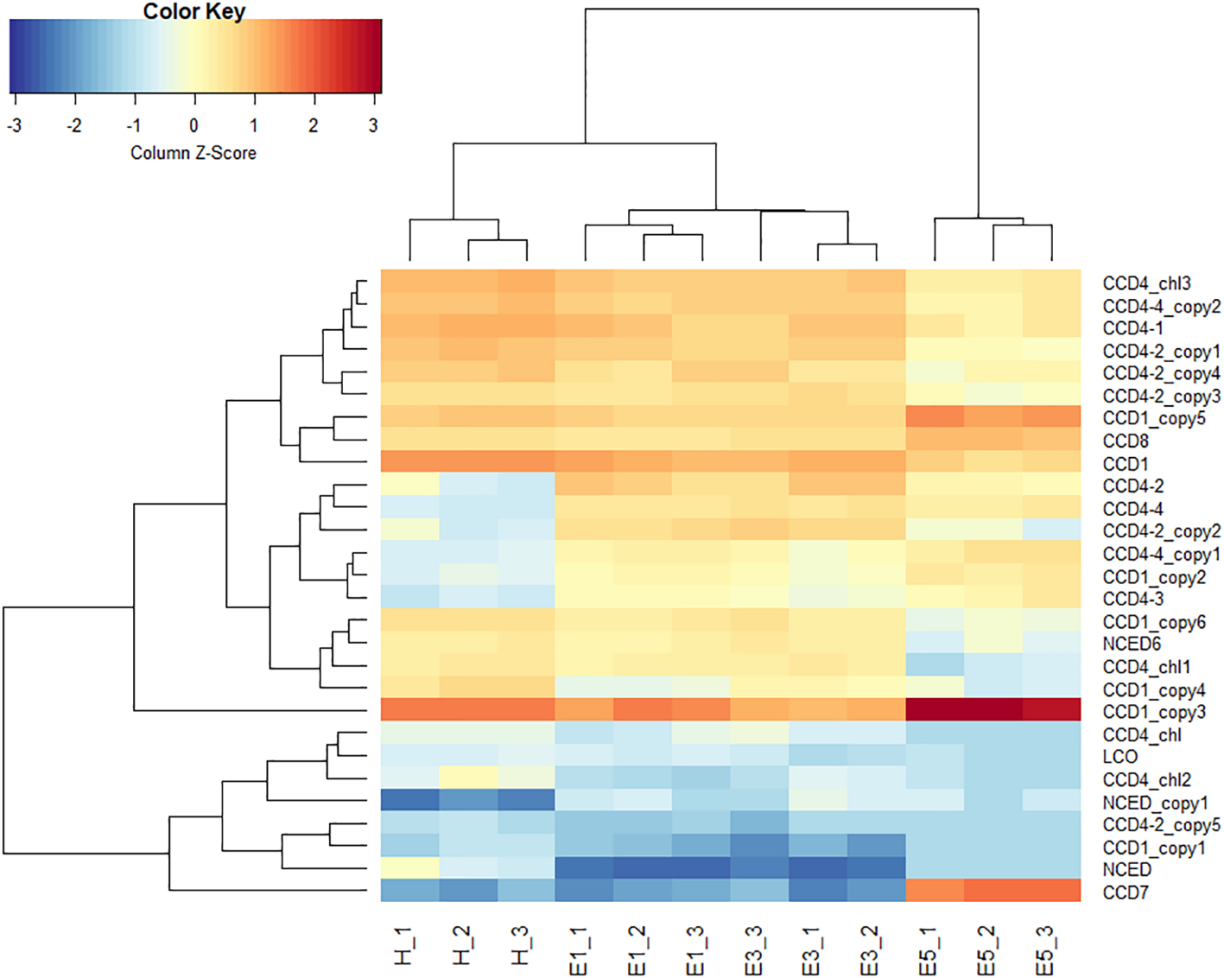
Transcriptomic expression profiling of *CCD* genes in leaves and seeds of *B. orellana*. The heatmap displays Z-score–normalized logCPM expression values of 28 *CCD*-related transcripts across seed (H1–H3) and leaf developmental stages (E1, E3, and E5; three biological replicates each). Red indicates higher relative expression and blue lower expression. Hierarchical clustering reveals distinct expression modules, with several *CCD1* and *CCD4* transcripts enriched in seeds, while specific *NCED* and other paralogs show preferential expression in leaf tissues. Gene names follow the nomenclature in **Table 2**. All 28 curated CCD protein sequences returned tBLASTn matches in the TSA database; however, five genes showed lower-confidence matches (identity < 70% and/or E-value > 0.01): *CCD8* (g24592.t1; 44.1% identity), *CCD7* (g38864.t1; 48.7%), *CCD4_chl1* (g33765.t1; 41.3%), LCO (g37269.t1; 54.4%), and *CCD4-4_copy2* (g33764.t1; 66.7%); expression values for these genes should be interpreted with caution. The remaining 23 genes showed high-confidence matches (identity ≥ 70%, E-value < 0.01), with most exhibiting 98–100% sequence identity. Read counts were log2-CPM normalized using edgeR v3.40 and visualized as a column Z-score scaled heatmap. Complete TSA match details are provided in **Supplementary Table 7**.

To confirm the transcriptional support of the genomic identification of *CCD* genes and refine their expression profiles, we expanded the transcriptomic analysis using the publicly available *B. orellana* Transcriptome Shotgun Assembly (TSA) and RNA-seq data from seed (H1–H3) and leaf developmental stages (E1, E3, and E5). A tBLASTn search of the 28 identified CCD protein sequences against the TSA database yielded high-confidence matches for 28 curated *CCD*-related transcripts in the TSA dataset, including multiple members of the *CCD1*, *CCD4*, *NCED*, *CCD8*, and *LCO* groups. Most matches exhibited high sequence identity (98–100%), confirming their transcriptional support (**Supplementary Table 7**). Of these, 23 returned high-confidence matches (sequence identity ≥ 70%, E-value < 0.01), with most exhibiting identity of 98–100%. The remaining five genes; *CCD8* (44.1% identity), *CCD7* (48.7%), *CCD4_chl1* (41.3%), *LCO* (54.4%), and *CCD4-4_copy2* (66.7%), showed lower-confidence matches; their expression values are included in the analysis but should be interpreted with caution given the reduced alignment quality. Expression profiling across tissues and developmental stages revealed marked tissue-specific and stage-dependent patterns (**Figure 7**). Several *CCD1* and *CCD4* members, including *CCD1_copy5*, *CCD4-3*, *CCD4-1*, and *CCD4-copy* variants, clustered together and displayed relatively higher expressions in seed samples compared to leaf tissues, consistent with a predicted role in apocarotenoid (bixin) biosynthesis during seed development and supporting their prioritization as candidates for future functional validation. In contrast, certain paralogs, such as *CCD4-2_copy5*, *CCD1_copy1*, and *CCD4_chl2*, showed reduced expression in seeds and relatively higher or stage-specific expression in leaves, suggesting possible functional diversification. *NCED* (g2824.t1) exhibited strong upregulation in late leaf stage E5, consistent with its predicted association with abscisic acid biosynthesis pathways. The *LCO* transcript and some *CCD1* and *CCD8* showed overall low expression across tissues, consistent with their lower-confidence TSA matches and possible limited expression in the sampled tissues. Unsupervised hierarchical clustering separated seed-enriched *CCD1* and *CCD4* transcripts from leaf-biased *NCED* and other paralogs, indicating co-expression patterns among specific *CCD* subfamilies during seed maturation. These results provide expression-based evidence that distinct *CCD1* and *CCD4* members are preferentially expressed in seeds, positioning them as strong candidates for participation in bixin biosynthesis, while other paralogs are predicted to fulfill broader roles in carotenoid turnover and hormone-related metabolism. However, it should be noted that elevated seed expression is consistent with but does not demonstrate a biosynthetic function, and direct functional validation remains necessary to confirm the roles of these candidates in the bixin pathway.

## 4 Discussion

### 4.1 Genome-wide identification and structural and phylogenetic characterization of the *CCD* gene family

The genome-wide analysis presented here successfully identified and characterized 28 non-redundant *CCD* genes in the *B. orellana* genome. Our dual-approach domain analysis confirming the presence of conserved catalytic motifs (e.g., the iron-binding histidine tetrad and dioxygenase catalytic signature) and Pfam domains (PF03055, PF10543) characteristic of the *CCD*/*RPE65* superfamily (**Supplementary Table 2**), confirmed the identification and provides a reliable foundation for understanding the molecular basis of apocarotenoid formation in this economically important species. The identification of 28 members aligns with findings in other specialized plants, where gene family sizes are often shaped by species-specific metabolic demands, as observed in *Nicotiana tabacum* (19 *CCDs*) (Zhou et al., 2019) and in *Brassica napus* (30 *BnCCD*) (Zhou et al., 2020). Our findings reveal a gene family with substantial structural diversity (**Table 1**), a feature that likely underpins functional specialization. The confirmation of key iron-binding histidine tetrads and dioxygenase signatures in all 32 high-confidence candidates supports their predicted functional annotation as bona fide carotenoid-cleaving enzymes and suggests their potential involvement in the initial steps of bixin biosynthesis from lycopene, as previously proposed (Bouvier et al., 2003; Us-Camas et al., 2022). Histidine density analysis showed that CCD proteins contained 1.36–3.8% histidine residues, further supporting their identity as members of the CCD/RPE65 metalloenzyme superfamily, a characteristic consistently reported in genome-wide studies of this family (Zhou et al., 2019).

The genomic architecture of *B. orellana CCD* genes revealed three broad structural categories, as we identified from phylogenetically organized gene structure analysis (**Figure 3**). The first encompasses genes with simple to intronless architectures (e.g., g37263.t2, g37263.t1, g2824.t1, g25911.t1, g37262.t2, g19008.t1, g19004.t1), which likely represents retrotransposition-derived paralogs, a phenomenon documented in the *CCD* families of *Brassica napus* and *Nicotiana* species, suggesting a common evolutionary mechanism for the rapid generation of structural and potentially functional diversity within this gene family across different plant lineages (Zhou et al., 2019, 2020). Mean pairwise Ks values for this group ranged from 0.452 (g37263.t1) to 0.906 (g37262.t2), reflecting moderate to high synonymous divergence consistent with their proposed origin through retrotransposition followed by sequence divergence. A second group displays intermediate two-to-three exon organizations (e.g., g24592.t1, g37269.t1, g38864.t1, g31486.t1, g33765.t1, g37259.t1, g37262.t1, and g37270.t1), with mean pairwise Ks values ranging from 0.453 (g37269.t1) to 1.265 (g37259.t1). Within this group, g24592.t1 (*CCD8*; Ks = 0.463) and g37269.t1 (*LCO*; Ks = 0.453) showed the lowest synonymous divergence, suggesting more recent divergence or stronger selective constraint relative to the other members. In contrast, g24592.t1 (Ks = 1.265) and g38864.t1 (Ks = 0.925) exhibited the highest Ks values, hinting at earlier duplication events or relaxed selective constraint in these lineages. The third and most structurally complex group, comprising g39248.t1, g23079.t1, g17637.t1, g17631.t1, g17629.t1, g17625.t1, and g17630.t1 is characterized by highly fragmented architectures with 10–15 or more short exons interspersed across transcripts that extend up to 6 kb. Mean pairwise Ks values for this group ranged from 0.367 (g17637.t1) to 1.449 (g23079.t1). Notably, g17637.t1 and g17625.t1 showed the lowest Ks values (0.367 and 0.594, respectively), consistent with stronger selective constraint on these core *CCD1* members, likely reflecting their role in essential or tightly regulated steps of carotenoid cleavage in *B. orellana*.

The higher Ks values observed for g23079.t1 (1.449) and g17629.t1 (0.913) suggest earlier divergence or relaxed constraint among the more peripheral *CCD1* paralogs. A particularly informative outlier group comprises g33764.t1, g37264.t1, and g37264.t2, which cluster separately in the dendrogram. Among these, g33764.t1 carried the highest mean Ks value in the entire dataset (1.244), followed by g37264.t2 (Ks = 0.714), both paired with divergent structural features, a single-exon and a three-exon architecture with two prominent introns, respectively. In contrast, g37264.t1 (Ks = 0.452) showed moderate divergence despite its nearly intronless architecture, suggesting that structural simplification and synonymous divergence have proceeded independently in these paralogs. Taken together, the Ks-based divergence patterns across all three structural categories indicate that the *B. orellana CCD* family has undergone extensive and heterogeneous evolutionary dynamics, with no single structural class showing uniformly low synonymous divergence, and that selective pressures have varied substantially among subfamilies and individual paralogs (**Supplementary Table 4**).

This structural variation implies diverse regulatory capacities: the multi-exon genes of the complex clade are prime candidates for alternative splicing-mediated regulation, potentially enabling context-specific production of *CCD* isoforms during bixin biosynthesis, whereas structurally simpler genes may fulfill more constitutive roles or have undergone neofunctionalization following structural reduction. Complementing the structural analysis, the GLAM2 motif search identified a highly conserved 48–50 amino acid motif present in 21 out of 28 curated CCD proteins (**Supplementary Figure 3**). This semi-conserved block, featuring critical residues such as tryptophan, phenylalanine, aspartate, and glycine, as reflected by the recurring FDGDG-type pattern, likely constitutes a core catalytic or substrate-binding region essential for carotenoid cleavage activity. The high conservation of this motif within the clade of structurally complex genes, despite the heterogeneous mean pairwise Ks values observed across this group (0.367–1.449), underscores the evolutionary constraint to maintain the enzymatic core has been maintained independently of synonymous divergence rates, suggesting that purifying selection acts primarily at the protein functional level rather than uniformly across the coding sequence. The synteny analysis (**Supplementary Figure 5**) provides additional evolutionary context for the genomic reorganization of the *B. orellana CCD* family. Partial conservation of flanking gene order with *A. thaliana* and *T. cacao* orthologs was observed for *CCD1*, *CCD7*, *CCD8*, and *NCED* subfamilies, consistent with their conserved roles in strigolactone and ABA biosynthesis across the Malvales and Brassicales. In contrast, the *CCD4* cluster at the distal end of scaffold 9 displayed no detectable inter-species synteny, and the *LCO* member (g37269), physically embedded within this cluster, showed no flanking gene conservation with either reference species, suggesting lineage-specific repositioning or origin. This pattern is consistent with the broader observation that genomic regions undergoing active tandem duplication tend to accumulate structural rearrangements that erode synteny while generating functional diversity (Erb and Kliebenstein, 2020; Li et al., 2021). Together, these results confirm that while the enzymatic core of the CCD/RPE65 superfamily is broadly conserved across angiosperms at the sequence level, the genomic context of *B. orellana CCD* loci has been substantially reorganized through lineage-specific tandem duplication and rearrangement, particularly within the *CCD4* subfamily, reinforcing the conclusion that this genomic hotspot on scaffold 9 underlies the metabolic specialization driving bixin production in achiote.

The formal paralog pair analysis (**Supplementary Figure 6**; **Supplementary Table 5**) provides quantitative support for these conclusions. Applying criteria of physical co-localization (same scaffold, distance < 200 kb) and synonymous divergence (Ks < 1.0), 39 tandem duplication candidate pairs were identified across the two principal genomic hotspots. The lower mean Ks of the *CCD1* array on scaffold 4 (0.162) relative to the *CCD4* cluster on scaffold 9 (0.403) suggests that *CCD1* expansion is more recent, while the broader Ks range and shorter inter-gene distances of the *CCD4* cluster are consistent with successive rounds of duplication and divergence over a longer evolutionary period. Three paralog pairs with Ks = 0 represent the most recently duplicated copies, while five *CCD4* pairs with Ks ≥ 1.0 despite distances < 200 kb indicate earlier events where synonymous sites have approached saturation. This pattern of heterogeneous synonymous divergence within confined genomic regions is characteristic of active duplication hotspots, as documented in expanded gene families of other specialized plant species (Li et al., 2021; Zhou et al., 2019).

### 4.2 Evolutionary diversification and putative functional implications of *CCDs*

The evolutionary history of the *CCD* family in achiote plant is marked by significant expansion and diversification, particularly within the *CCD1* and *CCD*4 subfamilies, which are directly implicated in bixin biosynthesis (Carballo-Uicab et al., 2019; Us-Camas et al., 2022). The maximum-likelihood phylogenetic cladogram, incorporating reference orthologs from *A. thaliana* and *T. cacao* and visualized in a circular layout, clearly delineated the subfamily classification (*CCD1*, *CCD4*, *CCD7*, *CCD8*, *NCED*, and *LCO*) and revealed a significant species-specific expansion within the *CCD1* and *CCD4* subfamilies (**Figure 2**), confirming that *B. orellana* possesses an expanded repertoire of *CCD* genes compared to *A. thaliana*, with notable species-specific clusters of *CCD1* and several *CCD4* isoforms. Furthermore, subcellular localization predictions using WoLF PSORT revealed distinct compartmentalization patterns among *BoCCD* members, potentially reflecting functional specialization. Most *B. orellana* CCD1 and core CCD4 proteins were predicted to localize in the cytoplasm, consistent with their proposed roles in apocarotenoid formation. Intriguingly, several *CCD1* isoforms (*CCD1_copy3*, *CCD1_copy4*, and *CCD1_copy5*) were strongly predicted to target the peroxisome, while specific *CCD4* variants assigned to the *CCD4_chl* subgroup showed predicted predominant chloroplastic localization (**Table 2****).** This diversity in subcellular targeting suggests a complex spatial organization of apocarotenoid metabolism, potentially channeling substrates and products between different organelles to optimize bixin biosynthesis or facilitate other physiological functions. The clustering of multiple *CCD1* and *CCD4* genes in distinct, well-supported clades suggests duplication events of varying antiquity, as reflected by the heterogeneous mean pairwise Ks values observed within both subfamilies (*CCD1*: 0.367–1.449; *CCD4*: 0.452–1.265; **Supplementary Table 4**). Members with lower Ks values, such as g17637.t1 (Ks = 0.367) within *CCD1* and g37264.t1 (Ks = 0.452) within *CCD4*, likely represent more recent duplications, while those with higher Ks values suggest earlier divergence event. This expansion aligns with patterns observed in other species with specialized apocarotenoid metabolism, such as *Crocus sativus* (Xu et al., 2024) and *Dendrobium officinale* (Wang et al., 2022), and underscores the pivotal role of lineage-specific diversification of gene families in the evolution of specialized metabolic pathways. In addition, the targeted similarity heatmap (**Figure 1**), focusing on the best-matched orthologs, confirmed robust orthology relationships between *A. thaliana* and *T. cacao*, facilitating functional annotation transfer. The phylogenetic placement of *B. orellana CCD1* genes alongside *A. thaliana AtCCD1* orthologs (O65572, A0A178V6B6; **Figure 2**) confirms a broadly conserved *CCD1* lineage across angiosperms, while the clustering of *B. orellana CCD4* members with both *A. thaliana* and *T. cacao CCD4* sequences highlights the shared evolutionary history of this subfamily within the Malvales and Brassicales orders. This suggests potentially conserved biochemical pathways for apocarotenoid metabolism in this lineage. This expansion of *CCD*s in species with high apocarotenoid content underscores the putative pivotal role of this gene family in generating diverse compounds for plant adaptation and human use (Xu et al., 2024; Wang et al., 2025). This diversification appears to be driven, at least in part, by tandem duplication events, as evidenced by the clustering of the *CCD1* gene on scaffold 4 and the large *CCD4* cluster at the distal end of scaffold 9 (**Figure 6**). The genomic colocalization of phylogenetically related *CCD1* and *CCD4* genes strongly suggests that tandem duplication has been a key mechanism for the expansion and functional diversification of subfamilies critical for bixin production. This expansion may provide the genetic raw material for metabolic specialization, potentially enabling the plant to fine-tune the production of specific apocarotenoids, such as bixin, in response to developmental or environmental cues (Erb and Kliebenstein, 2020). Such a link between gene duplication and functional specialization in secondary metabolism is a recurring theme, as demonstrated in the terpene synthase family of *Aquilaria sinensis*, where recent whole-genome duplication events have been associated with stress-induced sesquiterpene biosynthesis (Li et al., 2021).

**Figure 8:**
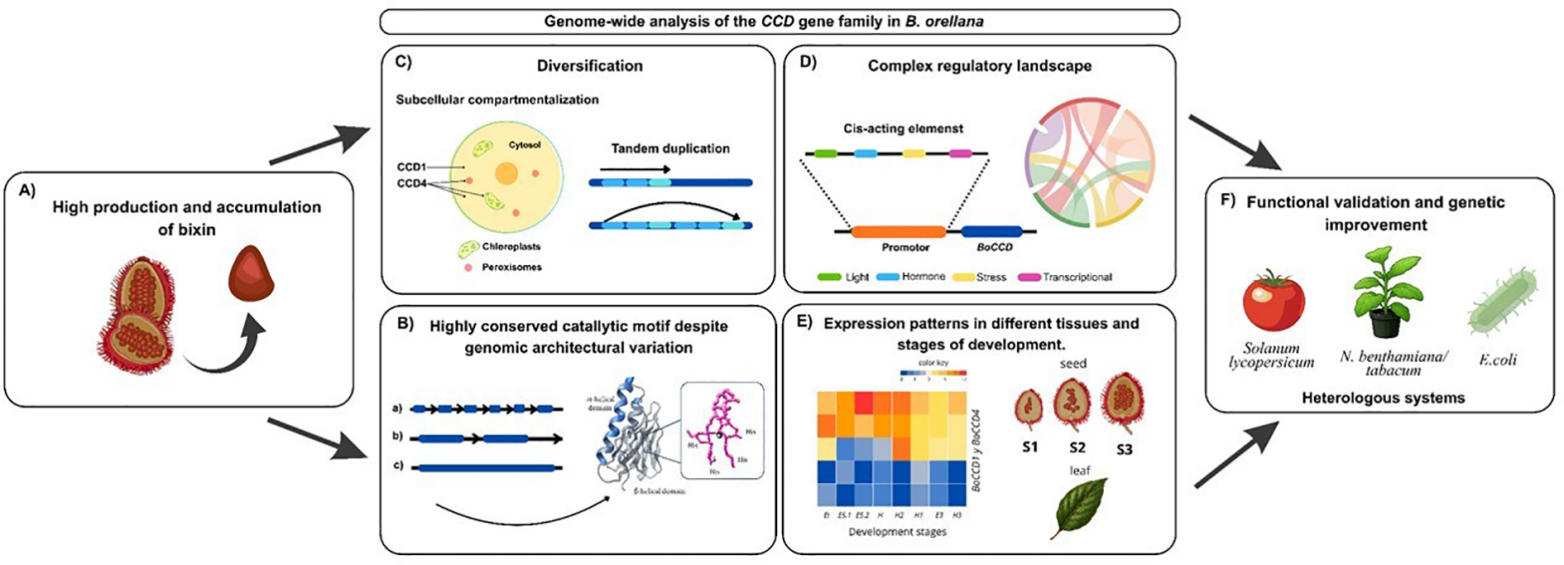
General overview of the first comprehensive genome-wide analysis of the *CCD* gene family in *B. orellana*. **(A)** Bixin production and accumulation occur predominantly in the seeds of *B. orellana* and are closely associated with *CCD* family members. **(B)** Genomic analysis revealed that, despite structural variation among genes, the catalytic site is highly conserved; the family displays marked structural diversity, organized into three phylogenetically defined architectural classes: (a) structurally complex genes with 10–15 or more short exons, (b) genes with intermediate structures containing 2–8 exons, and (c) intronless or single-exon genes, likely derived from retrotransposition events. **(C)** Strong evidence indicates that tandem duplication has been a key evolutionary force driving diversification of the *CCD* family, while subcellular compartmentalization (cytoplasm, chloroplasts, and peroxisomes) of apocarotenoid metabolism suggests functional specialization. **(D)** Several *CCD* members exhibit dense and diverse arrays of cis-regulatory elements, indicating potentially complex transcriptional regulation. **(E)** Transcriptomic profiling reveals tissue-specific expression patterns, particularly among *CCD1* and *CCD4* members. **(F)** Taken together, the prioritized *CCD* candidate genes identified in this study represent valuable targets for future functional validation and genetic improvement strategies using bacterial and plant heterologous systems. S1: stage one, S2: stage two, S3: stage three.

Promoter analysis (**Figures 4**, **5**) revealed a predicted regulatory potential landscape for the *CCD* family. The prevalence of *cis*-elements associated with light (e.g., G-box, Box 4), multiple hormones (ABRE for ABA; CGTCA-motif/TGACG-motif for MeJA; P-box/GARE-motif for gibberellins; TCA-element for salicylic acid), and various stresses (ARE, TC-rich repeats, LTR) indicates that the expression of these genes may be subject to sophisticated transcriptional modulation, as inferred from promoter sequence features alone. The abundance of ABA- and MeJA-responsive elements is particularly noteworthy, as these hormones are key regulators of *CCD* gene expression in other species (Zhou et al., 2019). Furthermore, the presence of stress-responsive elements, such as TC-rich repeats and MBS, suggests that *CCD* expression may be modulated by abiotic stresses, such as drought, a regulatory pattern observed in *Nicotiana* species (Zhou et al., 2019) and in tomato (Wei et al., 2016). The enrichment of light-responsive elements is particularly intriguing, given the role of *CCD*s in producing pigments and signaling molecules. The curved links in **Figure 5** connect genes sharing similar promoter element composition within each functional category, reflecting motif profile similarity rather than demonstrated co-regulation. This promoter architecture similarity potentially involving transcription factors from families such as MADS and MYB, as suggested by co-expression networks in *Prunus armeniaca* on *CCD*s (Zhang et al., 2019), is consistent with the hypothesis that environmental and developmental signals could converge to modulate the expression of genes linked to the bixin biosynthetic pathway, optimizing pigment accumulation in seeds. Indeed, the central role of transcription factors in integrating stress and hormone signals has been recently highlighted as a key mechanism bridging biotic and abiotic responses in plants (Liu et al., 2024). It should be noted, however, that PlantCARE-based identification of cis-elements reflects predicted regulatory potential inferred from sequence features and does not constitute direct evidence of transcriptional activity, factor binding, or regulatory causality; experimental validation would be required to confirm these inferences. In line with these observations, the transcriptomic expression analysis (**Figure 7**) provides expression-level context for selected *CCD* genes by revealing tissue-specific expression patterns. The elevated expression of specific *CCD1* and *CCD4* members (e.g., *CCD1-copy5*, *CCD4-3*, *CCD4-1*, and several *CCD4–2 copy* variants) in seed tissues is consistent with their predicted role in bixin biosynthesis, consistent with previous reports of *CCD* expression during seed development in achiote (Carballo-Uicab et al., 2019; Us-Camas et al., 2022). This connection is reinforced by existing functional evidence: g19008.t1 (*CCD4-1*) and g37262.t1 (*CCD4-4*) share 100% identity with *BoCCD4–1* and *BoCCD4-4*, both demonstrated to produce bixin aldehyde and norbixin from lycopene in *E. coli* (Us-Camas et al., 2022), providing direct support for their predicted biosynthetic role (**Supplementary Figure 7**). g37270.t1 (*CCD4-3*) likewise shares 100% identity with *BoCCD4-3*, whose heterologous characterization has revealed a broad substrate profile: lycopene cleavage at 5,6/5′,6′ was reported in *E. coli* (Carballo-Uicab et al., 2019), while cleavage at 7,8/7′,8′ yielding crocetin dialdehyde was demonstrated in *N. benthamiana* and *S. lycopersicum* (Frusciante et al., 2022), indicating wider regioselectivity than initially described and highlighting the value of multi-system validation. g37268.t1 (*CCD4-2*) shares 92.42% identity with the functionally validated *BoCCD4-2* (Us-Camas et al., 2022), (normalized WAG-model distance, **Table 2**; a direct ClustalW pairwise alignment yields a higher value of 99.43%, 519/522 aligned positions) but the 7.58% divergence may affect substrate specificity, warranting independent characterization. For the *CCD1* members (g17625.t1–g17637.t1, g23079.t1, g39248.t1), identity with *BoCCD1-1*, *BoCCD1-3*, and *BoCCD1–4* ranges only from 40% to 46%, precluding direct functional inference by homology. Moreover, all prior heterologous studies used sequences from the N4P accession, while the genome analyzed here corresponds to the P12 accession (Lieberman et al., 2026); inter-accession sequence divergence further reinforces the need for direct experimental validation of these *CCD1* paralogs. Beyond the seed-enriched candidates, paralogs such as *CCD4-2_copy5*, *CCD1_copy1*, and *CCD4_chl2* showing preferential expression in leaf tissues likely reflect subfunctionalization toward non-pigment metabolic roles, a pattern documented in the *CCD* family of *Crocus sativus* (Xu et al., 2024), though their enzymatic activities remain to be addressed. Some plastid-targeted members, including *CCD4-chl_copy1* and _copy2, and specialized subfamilies such as *NCED*, *CCD8*, and *LCO*, appear absent or lowly expressed in the *B. orellana* seed transcriptome (Cárdenas-Conejo et al., 2015), suggesting tissue-restricted roles whose contribution to carotenoid turnover or hormone-related metabolism cannot be excluded without further confirmation. The co-expression clustering of several seed-enriched *CCD* genes further suggests coordinated regulation, potentially facilitating the channeling of substrates toward bixin production. This coordinated expression, combined with the functional support provided by heterologous studies for key *CCD4* members and the identification of novel paralogs as priority targets for future validation, collectively points to a multifaceted genetic system underlying bixin accumulation in *B. orellana* seeds. Therefore, it is suggested that the diversification of the *CCD* family through gene duplication and its complex regulatory architecture, encompassing seed-specific expression, inter-accession allelic variation, and the catalytic versatility revealed by heterologous characterization of key members (Carballo-Uicab et al., 2019; Us-Camas et al., 2022; Frusciante et al., 2022), are key evolutionary adaptations that support the high-level, regulated production of bixin in *B. orellana* (**Figure 8****).**

The genomic framework established here, integrating structural diversity, phylogenetic expansion, promoter complexity, transcriptomic profiling, and codon-based divergence analysis, provides a comprehensive foundation from which targeted functional validation experiments can be designed to resolve the precise enzymatic contributions of individual *B. orellana CCD* members to the bixin biosynthetic pathway.

## 5 Conclusion

This study provides the first comprehensive genome-wide analysis of the *CCD* gene family in *B. orellana*, a species renowned for producing the apocarotenoid pigment bixin. We identified 28 non-redundant *CCD* genes, revealing a family marked by significant structural diversity, organized into three distinct architectural classes: (i) intronless or single-exon genes putatively derived from retrotransposition events, (ii) genes with intermediate 2–8 exon organizations, and (iii) structurally complex genes harboring 10–15 or more short exons spanning up to 6 kb. Codon-based Ka/Ks analysis revealed heterogeneous synonymous divergence rates (mean pairwise Ks: 0.367–1.449), with core *CCD1* members showing the lowest divergence values, consistent with stronger selective constraint, while the identification of a highly conserved catalytic motif (~48–50 aa) via GLAM2 and 10 conserved sequence motifs by MEME-based analysis underscore a strong evolutionary constraint on the enzymatic core of the CCD/RPE65 superfamily, suggesting that purifying selection acts primarily at the protein sequence level despite considerable genomic architectural variation. Phylogenetic and orthology analyses, supported by targeted similarity heatmaps and a circular maximum-likelihood phylogenetic cladogram incorporating reference orthologs from *A. thaliana* and *T. cacao*, supported the classification of these genes into the *CCD1*, *CCD4*, *CCD7*, *CCD8*, *NCED*, and *LCO* subfamilies, with a notable expansion observed in the *CCD1* and *CCD4* subfamilies. Subcellular localization predictions suggested potential diversification through cytoplasmic, peroxisomes, and chloroplastic targeting among different *CCD* members, indicating possible compartmentalization of apocarotenoid metabolic steps that awaits experimental confirmation.

The genomic organization of these genes, particularly the tandem array of *CCD1* members on scaffold 4 and, the large *CCD4* cluster at the distal end of scaffold 9, is formally supported by paralog pair analysis identifying 39 tandem duplication candidate pairs across these two hotspots. The lower mean Ks of the *CCD1* array (0.162) relative to the *CCD4* cluster (0.403) suggests more recent *CCD1* expansion, while three paralog pairs with Ks = 0 represent the most recently duplicated copies, collectively providing quantitative evidence that tandem duplication is the primary driver of B*. orellana CCD* family diversification. Synteny analysis with *A. thaliana* and *T. cacao* confirmed partial conservation of genomic neighborhoods for *CCD1*, *CCD7*, *CCD8*, and *NCED* subfamilies, and the lineage-specific nature of the *CCD4* expansion on scaffold 9. Furthermore, promoter analysis uncovered a predicted putative regulatory potential responsive to light, hormones and abiotic stresses based on promoter sequence features, with the *CCD1* and *CCD4–2* subfamilies exhibited particularly dense putative cis-elements arrays. Transcriptomic expression profiling revealed seed-enriched expression of *CCD1_copy5*, *CCD4-1*, *CCD4-3*, and multiple *CCD4–2* copy variants, consistent with their predicted role in bixin biosynthesis, while other paralogs showed preferential leaf expression, suggesting subfunctionalization. Our findings lay a crucial genomic foundation for understanding apocarotenoid metabolism in achiote as summarized in **Figure 8**. The expansion and diversification of the *CCD* family, together with the predicted transcriptional regulatory potential and possible subcellular compartmentalization inferred from genomic and bioinformatic analyses, likely contribute to the high levels of bixin production and accumulation in seeds, however, functional validation of individual candidates remains a key priority for future work. The candidate genes prioritized in this study represent valuable targets for such future functional validation and genetic improvement strategies to enhance bixin yield in *B. orellana*.

## Data Availability

The datasets presented in this study can be found in online repositories. The names of the repository/repositories and accession number(s) can be found in the article/**Supplementary Material**.
