# Supplementary material for: Genome-wide identification and characterization of the carotenoid cleavage dioxygenase gene family in Bixa orellana L
Source: Front Plant Sci. 2026 Jul 7;17:1885876. doi: 10.3389/fpls.2026.1885876 (PMC13386687; doi:10.3389/fpls.2026.1885876)
Supplement: Supplementary file 1 [file DataSheet1.pdf]

Genome-Wide Identification and Characterization of the Carotenoid Cleavage Dioxygenase (*CCD*) Gene Family in *Bixa orellana* L.

Supplementary Figures

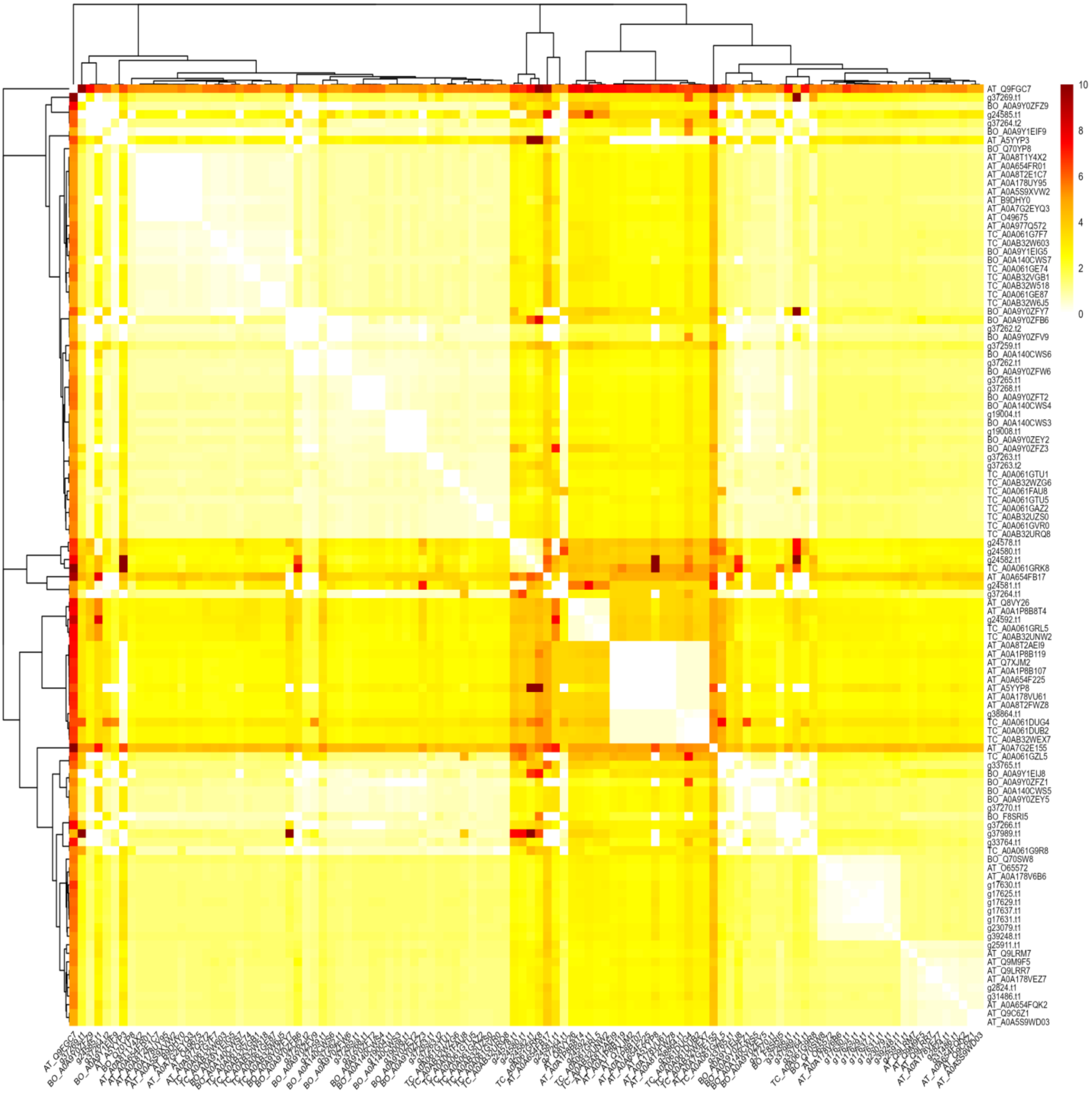

Supplementary Figure 1. Sequence similarity analysis of *CCD* gene family across species. Global sequence similarity heatmap displaying pairwise identity percentages among all carotenoid cleavage dioxygenase (*CCD*) genes from *Bixa orellana* (query sequences) and reference species (*Arabidopsis thaliana* and *Theobroma cacao*). Hierarchical clustering was performed using Euclidean distance, with color gradient from white (low similarity) to dark red (high similarity). The analysis includes the complete dataset of 66 reference sequences and reveals the overall similarity landscape and clustering patterns across the entire CCD gene family.

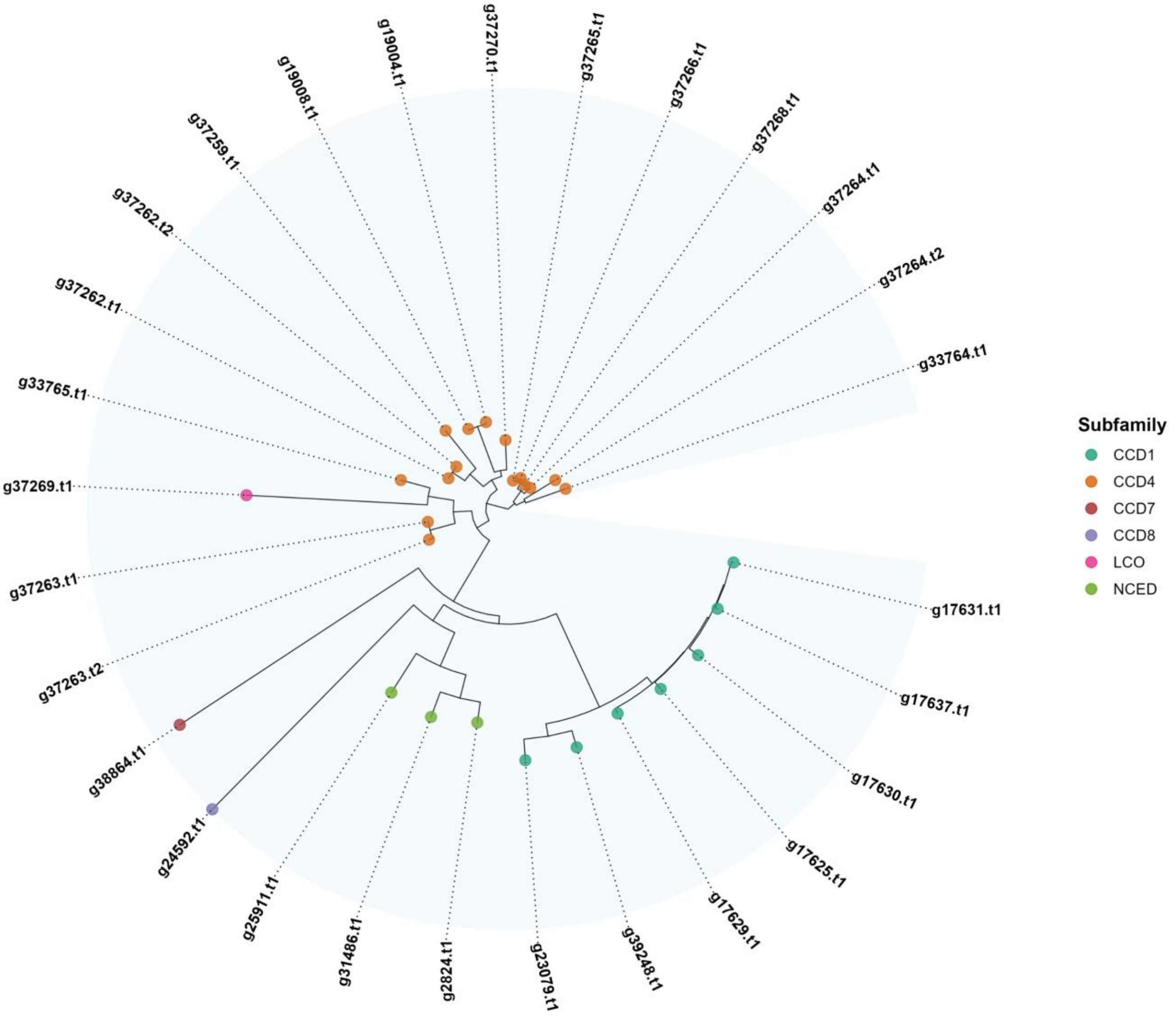

Supplementary Figure 2. Phylogenetic inference using an MRCA algorithm of *B. orellana CCD* genes. Tips are colored by subfamily classification (*CCD1*, *CCD4*, *CCD7*, *CCD8*, *LCO*, and *NCED*), with clades highlighted and labeled accordingly. Bootstrap values from 1000 replicates indicate branch support.

NAME	START	SITES	END	STRAND	MARGINAL
g24592.t1	92	LDVQGKIPLWLNGT.YLRNGPGLWHIEDYNFRHLFDGYATLVKLHFE.NG.RL	141	+	5.50
g31486.t1	141	LPIIGKIPECIQGV.YVRNGANPLHEPVAGH.HFFDGDGMVHAVRFH.NG.SA	189	+	125.
g2824.t1	123	LPVTGTIPACINGV.YLRNGANPLFEPIAGH.HFFDGDGMVHAVSID.NG.SA	171	+	115.
g25911.t1	93	LEVVGEIPVCLRGV.YVRNGANPLFAPSGGH.HLFDGDGMIHAVKLG.LGNKA	142	+	94.6
g33765.t1	35	APVEGKLPLSLDGV.YICNGLNPQHKPLSAL.HLSEGKVMIGRVDFR.KG.RG	83	+	36.1
g37268.t1	64	LIVEGEIPTSLDGAAYIRNGTNPQYIPDRAL.HFFEGDGMLHSLRFS.NG.RA	113	+	149.
g37265.t1	64	LIVEGEIPTSLDGAAYIRNGTNPQYIPDRAL.HFFEGDGMLHSLRFS.NG.RA	113	+	149.
g37266.t1	64	LIVEGEIPTSLDGAAYIRNGTNPQYIPDRAL.HFFEGDGMLHSLRFS.NG.RA	113	+	149.
g37263.t2	96	PVVKGELPSSLNGV.YIRNGPNPQHKPIGAL.HLFDGDGMLHSLQFS.NG.KA	144	+	145.
g37263.t1	96	PVVKGELPSSLNGV.YIRNGPNPQHKPIGAL.HLFDGDGMLHSLQFS.NG.KA	144	+	145.
g37270.t1	124	PVVEGQLPPSLNGTAYLRNGSNPQLRPRRAL.QYFEADGMIHSLHFSSDG.RA	174	+	92.5
g37262.t1	64	PVVEGELPSSLEGAAYIRNGPNPQFLPQRAL.HSFEGDGMLHSLRFS.NG.RA	113	+	134.
g19008.t1	44	CPIEGELPPSLHGAAYIRNGTNPQYKPQRAL.HIFEGDGMLHSLRFS.EGDRA	94	+	139.
g19004.t1	44	CPIEGELPPSLHGAAYIRNGTNPQYKPQRAL.HIFEGDGMLHSLRFS.EGDRA	94	+	139.
g39248.t1	71	LPVQGFLPECLNGE.FVRIGPNPKFHPVAGY.HWFDGDGMVHGLQIK.DG.KA	119	+	142.
g23079.t1	71	LSVRGYLPECLNGE.FVRIGPNPKFSPLSGY.HWFDGDGMVHGLRIK.DG.KA	119	+	133.
g17637.t1	72	LPVKGHLPECLNGE.FVRVGPNPKFAPVAGY.HWFDGDGMVHGMRIK.DG.KA	120	+	156.
g17631.t1	72	LPVKGHLPECLNGE.FVRVGPNPKFAPVAGY.HWFDGDGMVHGLRIK.DG.KA	120	+	157.
g17629.t1	72	LPVKGHLPECLNGE.FVRVGPNPKFAPVAGY.HWFDGDGMVHGMRIK.DG.KA	120	+	156.
g17625.t1	65	LPVKGHLPECLNGE.FVRVGPNPKFAPVAGY.HWFDGDGMVHGVRIK.DG.KA	113	+	156.
g17630.t1	66	LPVKGHLPECLNGE.FVRVGPNPKFAPVAGY.HWFDGDGMVHGMRIK.DG.KA	114	+	156.

Score: **2308.17**

logo with ssc

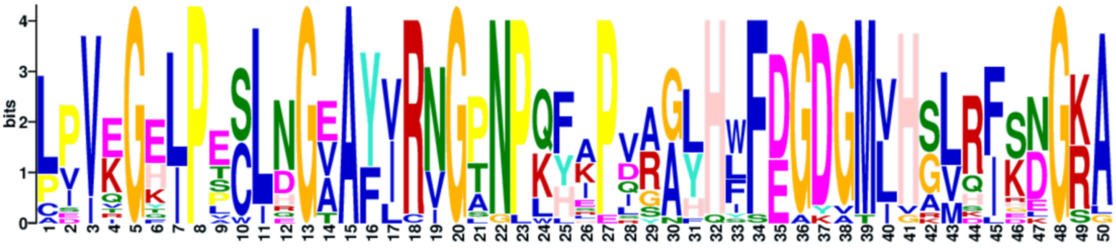

Score: 2235.49

logo with ssc

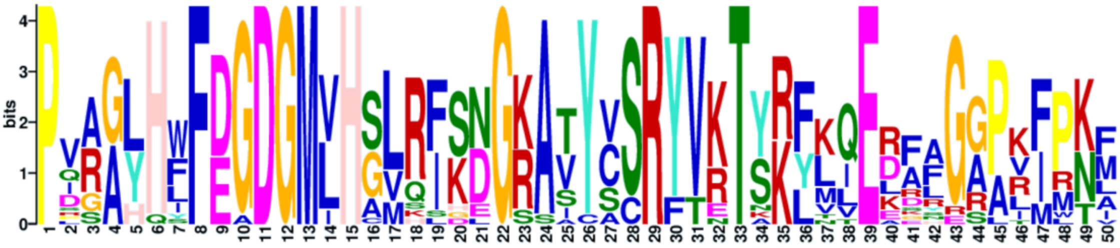

Score: 2160.64

logo with ssc

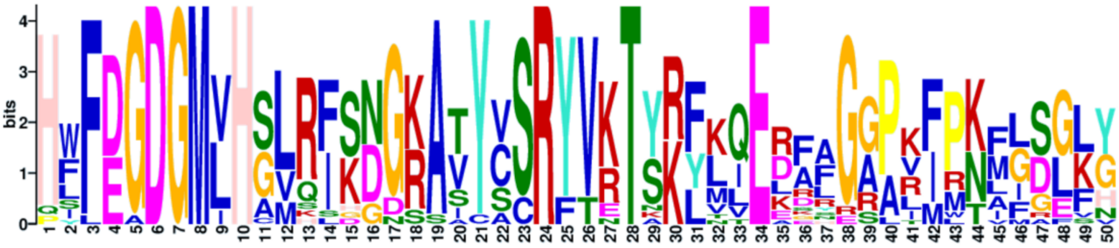

Supplementary Figure 3. GLAM2 motif alignment of the *B. orellana CCD* gene family. GLAM2 motif alignment of the 21 CCD protein sequences exhibiting the conserved motif that is important for carotenoid cleavage activity. The alignment panel (top) shows the identified motif site per sequence along with start/end positions and marginal scores; all sequences were recovered on the positive strand. Three sequence logos (bottom) generated at scores of 2308.17, 2235.49, and 2160.64 illustrate the semi-conserved motifs detected across the 50-position alignment. Letter heights in the logos correspond to the information content (bits) of each residue, highlighting conserved positions, particularly tryptophan, phenylalanine, aspartate, and glycine, within the putative catalytic region.

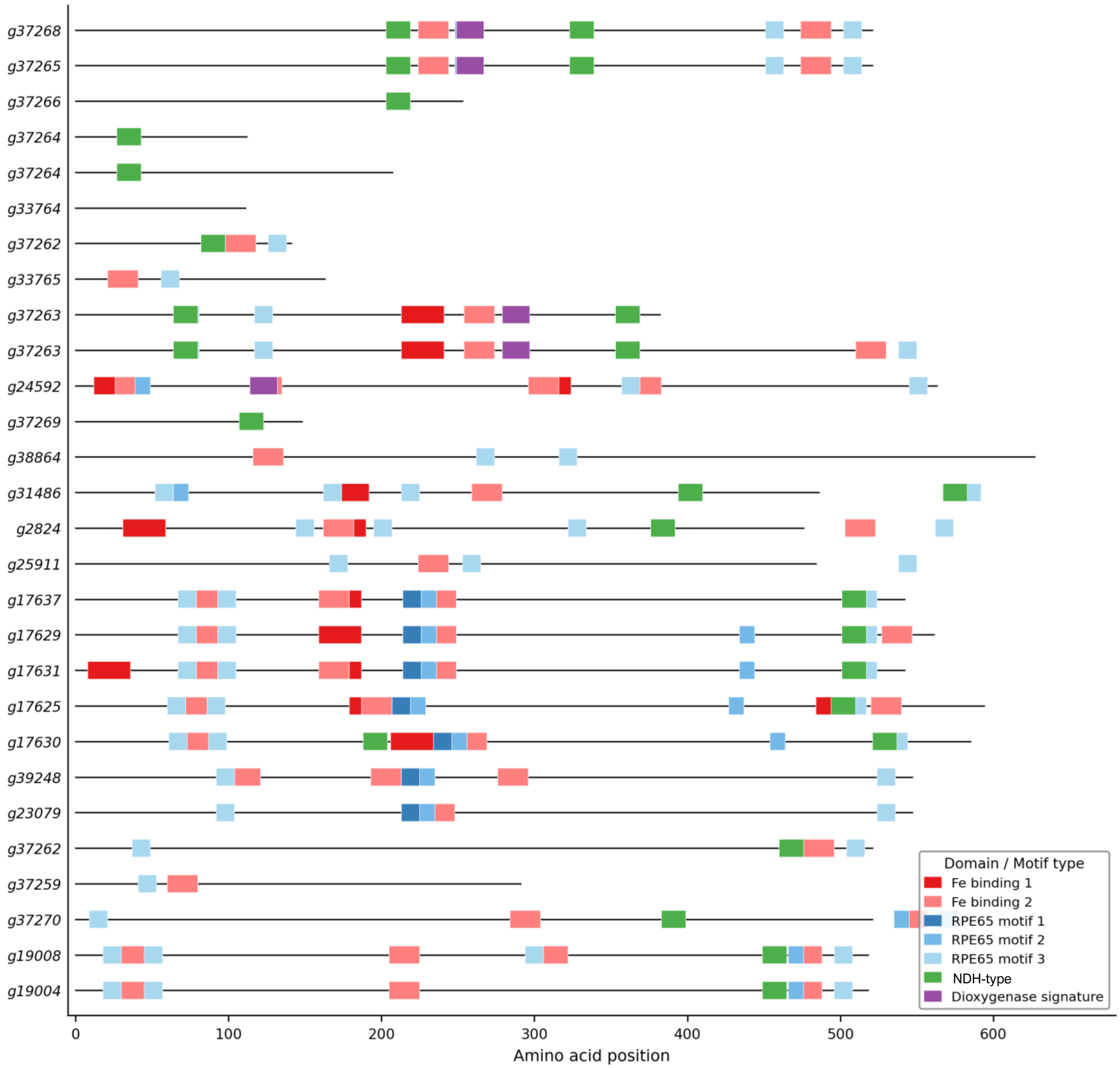

Supplementary Figure 4. Local domain and motif architecture of the 28 curated *Bixa orellana* CCD proteins, ordered according to the maximum-likelihood phylogenetic tree (Supplementary Figure 2). Each horizontal bar represents a protein sequence scaled proportionally to its length in amino acids. Colored rectangles indicate the position and type of each detected motif identified by regular-expression-based local searches: iron-binding histidine tetrads Fe_binding_1 ($H[X]_{20-40}H[X]_{20-40}H[X]_{20-40}H$, dark red) and Fe_binding_2 ($H[X]_{2-5}[DE][X]_{15-35}H$, light red); RPE65/CCD-specific motifs RPE65_motif_1 (dark blue), RPE65_motif_2 (medium blue), and RPE65_motif_3 (light blue); NDH-type motif (green); and dioxygenase catalytic signature (purple). Sequences with no detected motifs are shown as backbone lines only.

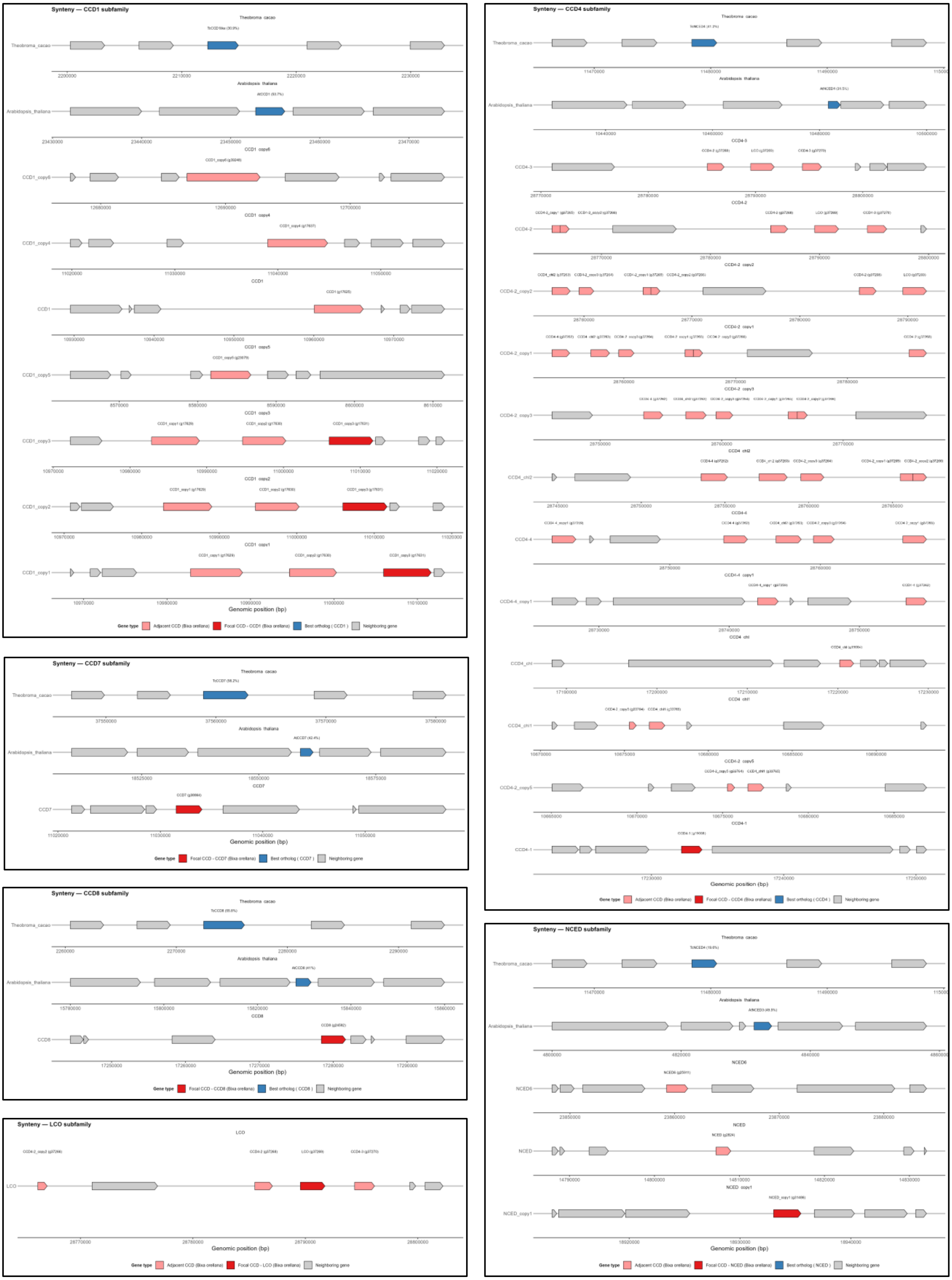

Supplementary Figure 5. Synteny conservation of *CCD* gene subfamilies in *B. orellana* relative to reference species. Genomic neighborhood comparison for each *CCD* subfamily, showing the focal *B. orellana CCD* gene (red) flanked by five adjacent *B. orellana CCD* genes (pink) and neighboring non-*CCD* genes (gray) on each side. The best-matched ortholog in *A. thaliana* and *T. cacao* is shown in blue for each subfamily, with its own flanking genomic context. Gene arrows indicate transcriptional orientation. Genomic positions are expressed in base pairs relative to the focal gene locus. Panels are organized by subfamily: *CCD1* (scaffold 4), *CCD4* (scaffold 9), *CCD7* (scaffold 10), *CCD8* (scaffold 5), *LCO* (scaffold 9), and *NCED* (scaffolds 1, 5, and 6). Ortholog identity percentages are indicated above each reference gene.

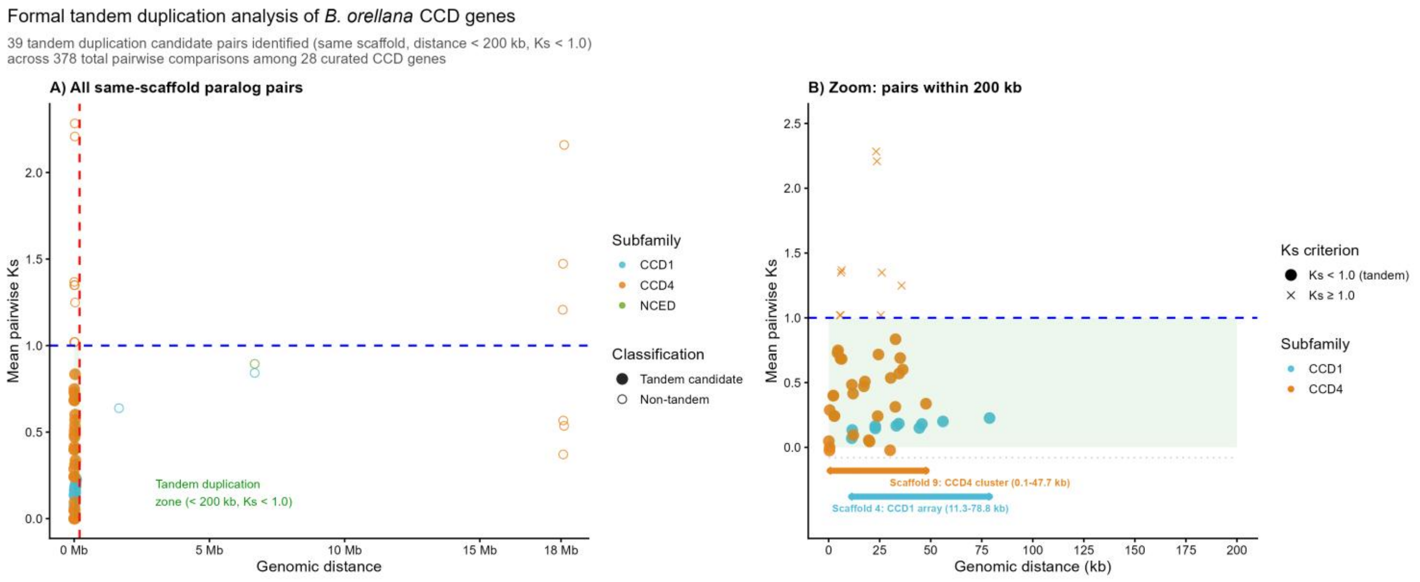

Supplementary Figure 6. Formal tandem duplication analysis of *Bixa orellana CCD* paralog pairs**.** Panel A shows mean pairwise Ks versus genomic distance for all 116 same-scaffold paralog pairs among the 28 curated *CCD* genes. Filled circles indicate tandem duplication candidates meeting all three criteria: same scaffold, genomic distance < 200 kb, and Ks < 1.0; open circles indicate pairs not meeting all criteria. The red dashed line marks the 200 kb distance threshold; the blue dashed line marks Ks = 1.0; the green-shaded region defines the tandem duplication zone. Panel B shows a zoom view of pairs within 200 kb, with × symbols indicating pairs exceeding the Ks threshold. Colored brackets indicate the two principal tandem arrays: the CCD4 distal cluster on scaffold 9 (orange; mean Ks = 0.403) and the *CCD1* array on scaffold 4 (blue; mean Ks = 0.162). In total, 39 tandem duplication candidate pairs were identified, supporting tandem duplication as the primary driver of CCD family expansion in *B. orellana* (Supplementary Tables 4 and 5).

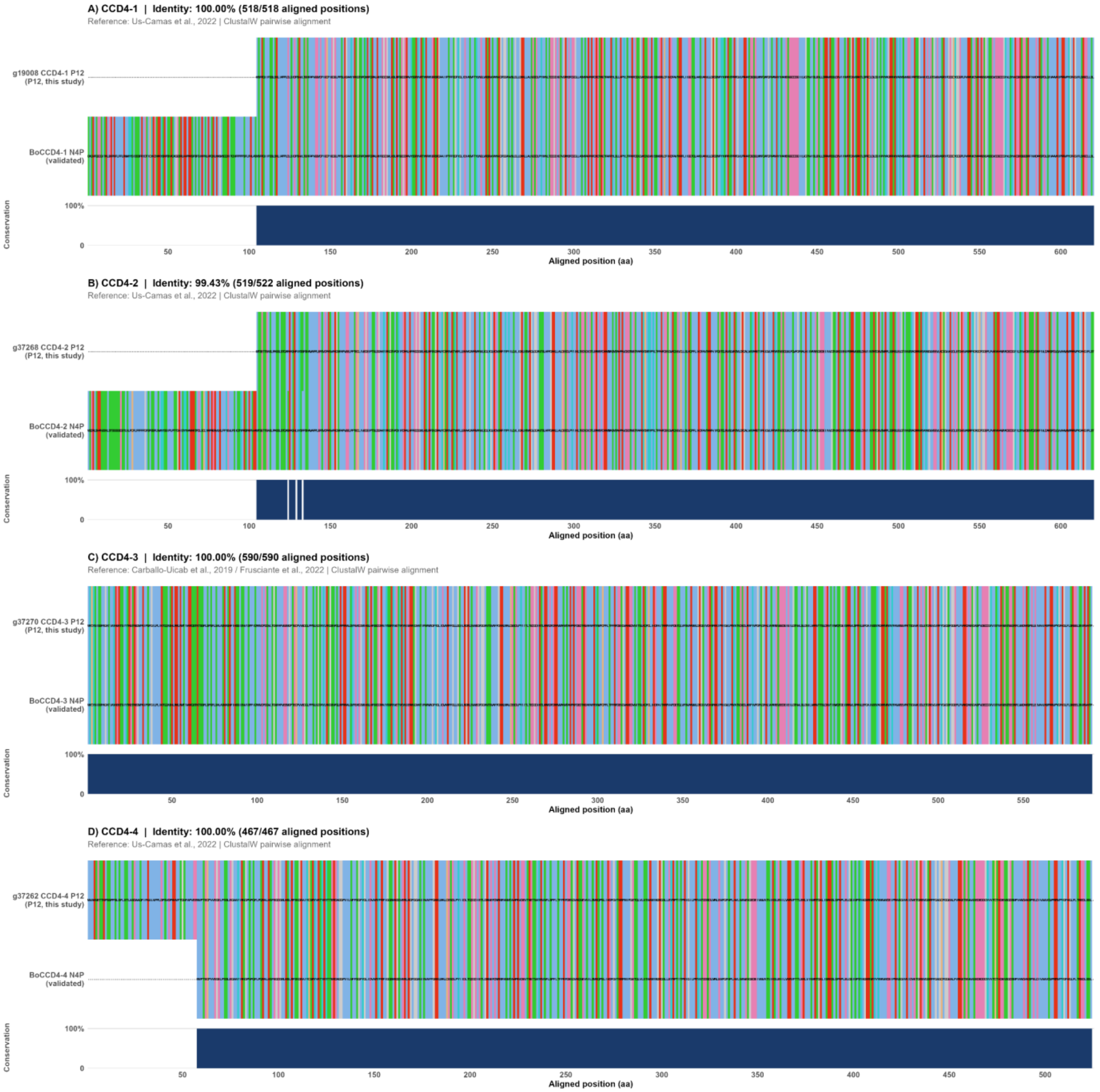

Supplementary Figure 7. Pairwise protein sequence alignments of *B. orellana CCD4* candidates identified in the P12 genome (this study) against previously validated *CCD4* sequences from the N4P accession. Each panel shows a ClustalW pairwise alignment between a study sequence (P12 genome, top) and its corresponding validated reference (N4P accession, bottom): A) g19008.t1 (*CCD4-1*) vs *BoCCD4-1* (Us-Camas et al., 2022); B) g37268.t1 (*CCD4-2*) vs *BoCCD4-2* (Us-Camas et al., 2022); C) g37270.t1 (*CCD4-3*) vs *BoCCD4-3* (Carballo-Uicab et al., 2019; Frusciante et al., 2022); D) g37262.t1 (*CCD4-4*) vs *BoCCD4-4* (Us-Camas et al., 2022). Each amino acid is colored according to the ClustalX physicochemical scheme: blue = hydrophobic (A, V, I, L, M, F, W, P); red = positively charged (K, R, H); magenta = negatively charged (D, E); green = polar (S, T, N, Q); tan = Cys; grey = Gly; cyan = Tyr; white = gap. The conservation histogram below each alignment indicates the proportion of identical positions per site (dark blue bars = 100% identity; grey bars = substitution or gap). Identity values were calculated as the number of identical positions relative to total non-gap aligned positions (ClustalW pairwise alignment): *CCD4-1* = 100% (518/518), *CCD4-2* = 99.43% (519/522), *CCD4-3* = 100% (590/590), *CCD4-4* = 100% (467/467). Note that these values differ from the inter-accession identity estimates reported in Table 2 of the main text, which were calculated using normalized pairwise protein distances under the WAG substitution model applied to the full dataset of 28 curated *CCD* sequences and all reference orthologs.
